# *In vitro* Evaluation of Isoniazid Derivatives as Potential Agents Against Drug-Resistant Tuberculosis

**DOI:** 10.3389/fphar.2022.868545

**Published:** 2022-05-04

**Authors:** Joaquim Trigo Marquês, Catarina Frazão De Faria, Marina Reis, Diana Machado, Susana Santos, Maria da Soledade Santos, Miguel Viveiros, Filomena Martins, Rodrigo F. M. De Almeida

**Affiliations:** ^1^ Centro de Química Estrutural, Institute of Molecular Sciences, Departamento de Química e Bioquímica, Faculdade de Ciências, Universidade de Lisboa, Lisboa, Portugal; ^2^ Instituto Superior de Educação e Ciências (ISEC Lisboa), Lisboa, Portugal; ^3^ Unidade de Microbiologia Medica, Global Health and Tropical Medicine, Instituto de Higiene e Medicina Tropical, Universidade Nova de Lisboa, Lisboa, Portugal

**Keywords:** antimycobacterial activity, *Mycobacterium tuberculosis*, isoniazid resistance, human serum albumin binding, cytotoxicity, lipophilicity

## Abstract

The upsurge of multidrug-resistant tuberculosis has toughened the challenge to put an end to this epidemic by 2030. In 2020 the number of deaths attributed to tuberculosis increased as compared to 2019 and newly identified multidrug-resistant tuberculosis cases have been stably close to 3%. Such a context stimulated the search for new and more efficient antitubercular compounds, which culminated in the QSAR-oriented design and synthesis of a series of isoniazid derivatives active against *Mycobacterium tuberculosis*. From these, some prospective isonicotinoyl hydrazones and isonicotinoyl hydrazides are studied in this work. To evaluate if the chemical derivatizations are generating compounds with a good performance concerning several *in vitro* assays, their cytotoxicity against human liver HepG2 cells was determined and their ability to bind human serum albumin was thoroughly investigated. For the two new derivatives presented in this study, we also determined their lipophilicity and activity against both the wild type and an isoniazid-resistant strain of *Mycobacterium tuberculosis* carrying the most prevalent mutation on the *katG* gene, S315T. All compounds were less cytotoxic than many drugs in clinical use with IC_50_ values after a 72 h challenge always higher than 25 µM. Additionally, all isoniazid derivatives studied exhibited stronger binding to human serum albumin than isoniazid itself, with dissociation constants in the order of 10^−4^–10^−5^ M as opposed to 10^−3^ M, respectively. This suggests that their transport and half-life in the blood stream are likely improved when compared to the parent compound. Furthermore, our results are a strong indication that the N′ = C bond of the hydrazone derivatives of INH tested is essential for their enhanced activity against the mutant strain of *M. tuberculosis* in comparison to both their reduced counterparts and INH.

## Introduction

As the 2030 deadline established by WHO to eradicate tuberculosis (TB) approaches, no significant advances concerning the mitigation of the disease have been achieved. Unfortunately, although TB is curable and preventable, the numbers associated to this disease are still quite alarming and, consequently, it remains as one of the top 10 causes of death worldwide and, until 2019, the main cause of mortality from a single infectious agent. The figures recently published in the WHO global TB report 2021 (which refers to the 2020 situation) (World Health Organization, 2021) show a practically immutable situation over the last few years up to 2019, that only changed dramatically in 2020 (7.1 million new case notifications of people newly diagnosed with TB in 2019 as opposed to 5.8 million in 2020) due to the high prevalence of COVID-19, which caused a disruption in the notification of new TB cases. The number of total cases per year, remains clearly above 10 million since 2000 (World Health Organization, 2021). An encouraging fact, on the other hand, was the progressive decrease of total deaths attributed to TB, which, in 2020, were around 1.3 million among HIV-negative people, a reduction of 0.6 million as compared to 2000. However, as compared to the 2019’s situation, there was an increase in the total number of TB-induced deaths. This scenario arose due to the significant decrease in TB treatment as a consequence of the much smaller number of TB cases detected and notified as a result of the COVID-19 pandemic (Ong et al., 2020; World Health Organization, 2021). Additionally, a rather preoccupying situation that might help explain the unsuccess in effectively controlling and reducing these numbers is the proliferation of multidrug-resistant tuberculosis (MDR-TB), a form of TB which is not countered by the two most powerful first-line antituberculars, isoniazid (INH) and rifampicin. In fact, ∼3% of new TB cases had MDR or rifampicin-resistant-TB (World Health Organization, 2021). Recently, two new drugs, Bedaquiline and Delamanid, have been introduced in a combination regimen to treat MDR-TB in adults, though without ensuring a side effect-free therapy (Brigden et al., 2015; Zumla et al., 2015). In 2019, another drug, Pretomanid, was approved by the United States FDA for the treatment of pulmonary treatment-intolerant or nonresponsive MDR-TB or extensively drug-resistant tuberculosis (XDR-TB) in adults (Keam, 2019).

The effectiveness of INH to fight TB is known since the 1950’s when its high activity against *Mycobacterium tuberculosis* (*Mtb*) was first described (Bernstein et al., 1952; Vilchèze and Jacobs, 2007). INH is a pro-drug that, when activated by the *Mtb* catalase-peroxidase enzyme KatG, leads to the inhibition of the biosynthesis of mycolic acids and, consequently, cell lysis (Winder and Collins, 1970; Heym et al., 1993; Rawat et al., 2003; Zhao et al., 2006; Vilchèze and Jacobs, 2007; Wiseman et al., 2010). Mutations in the *katG* gene represent the most frequent mechanism of resistance to INH (Hazbón et al., 2006). Thus, the overall picture of TB demands the development of new, effective and low toxicity antitubercular compounds and, within this scope, the design of new INH derivatives which can be an effective route to overcome *Mtb* drug resistance. With that in mind, we have previously described the QSAR-oriented design and synthesis of several new and more active INH derivatives with enhanced lethality against MDR-TB (Martins et al., 2014). Although the most active compound against the most frequent *katG* mutation, *i.e.*, the *katG* S315T mutant was a compound with a lengthy C10 alkyl chain, lipophilicity was not found as an important determinant of the antitubercular activity of these derivatives (Martins et al., 2014), neither the steric hindrance due to larger substituents seemed to be a ruling factor as regards the activity of the derivatives against the mutant strain (Teixeira et al., 2015). A recent lipophilicity assessment of a series of these isoniazid derivatives led to the conclusion that zwitterionic hydrazones partition to the outer palisade region of the sodium dodecyl sulfate micelles, whereas neutral hydrazides tend to penetrate further into the micellar core (Santos et al., 2020). INH, on its turn, shows a pH-dependent affinity for the membrane/water interface of a phospholipid bilayer (Pinheiro et al., 2014). Therefore, even though lipophilicity is not a factor influencing the activity of the compounds, it seems to promote an enhancement in their bioavailability inside the cell (Vila-Viçosa et al., 2017; Machado et al., 2018).

The selectivity index (SI) and the ability to bind plasma proteins are important parameters to predict the *in vivo* potency of a compound (Muller and Milton, 2012). SI translates quantitatively the relationship between the safety (toxicity) of a drug and its efficacy. It can help guiding the development of new drugs and a high SI is always desirable since it indicates a drug with a more favorable safety profile. However, in the case of life-threatening diseases for which no adequate treatment exists, drugs with low SI may also be acceptable (Muller and Milton, 2012).

On the other hand, the study of the interaction of a drug with plasma proteins may provide important insights concerning the pharmacokinetics (absorption, distribution, metabolism, and excretion—ADME processes) of a pharmaceutical agent within the body (Smith et al., 2010; Pellegatti et al., 2011; Wanat, 2020). The free drug hypothesis advocates that it is the free drug concentration, at the site of action, that exerts the biological activity, not the total drug concentration or the bound drug concentration to either plasma or tissue proteins (Smith et al., 2010). According to this hypothesis, envisaging drug candidates exhibiting reduced plasma protein binding is not the most fruitful strategy, since the binding to plasma proteins increases the solubility of the prospective pharmaceutical agent, extends its *in vivo* half-life by avoiding premature clearance, and slows down or prevents its passive extravasation to the target tissues. All of this has a remarkable impact on the bioavailability of the drug. In fact, the report on plasma protein binding is an FDA requirement in screening potential therapeutic agents.

Human serum albumin (HSA) is the most abundant protein in the plasma (35–50 mg/ml) (Wanat, 2020) and, although it plays a myriad of other functions, excels at being the most important non-specific transporter protein in the blood (Colmenarejo, 2003; Kanakis et al., 2006; Maiti et al., 2006; Pessoa and Tomaz, 2010; Schmidt et al., 2010; Kratz and Elsadek, 2012). HSA is comprised of a single chain containing three structurally similar domains termed I, II and III. In turn, each of these domains is constituted by two helical subdomains (A and B) (Curry et al., 1998; Ascenzi and Fasano, 2010). The crystal structure of HSA revealed the presence of binding sites for aromatic and heterocyclic ligands within subdomains IIA and IIIA, corresponding to Sudlow proposed sites I and II, respectively (Sudlow et al., 1976; Dockal et al., 1999; Petitpas et al., 2001).

**FIGURE 1:**
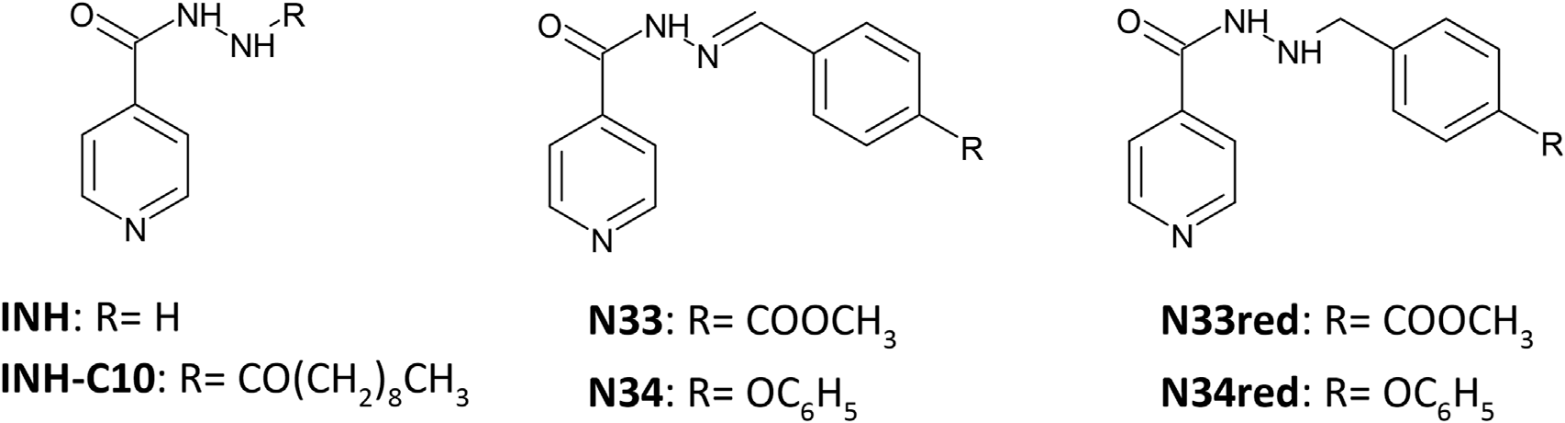
Structure of the compounds studied in this work.

Considering all the above, we provide a detailed biophysical study of the interaction of a series of INH derivatives—Figure 1 - with this plasma protein and a correlation study with the corresponding lipophilicity is undertaken. We also determined their cytotoxicity towards HepG2 Cells. From these assays, a SI was calculated considering the minimum inhibitory concentration (MIC) against both the wild type *Mtb* and the mutant S315T *katG Mtb*.

## Materials and Methods

### General

All reagents and solvents were obtained from commercial suppliers with analytical grade and used without further purification. HSA (lyophilized powder, ≥ 97% pure) was obtained from Merck (ref. A9511, Sigma-Aldrich^®^). The phosphate buffered saline (PBS: 0.01 M Na_2_HPO_4_, 0.0018 M KH_2_PO_4_, 0.0027 M KCl and 0.137 M NaCl, pH = 7.4) was prepared by dissolving pre-prepared tablets from VWR in MilliQ^®^ water (resistivity >18 MΩ cm). Chromatographic purification was performed on silica-gel 60, 0.004–0.066 mm, 230–400 mesh ASTM (Merck ref. 1.09385, Millipore^®^). Analytical thin layer chromatographies (TLC) were carried out on precoated silica gel 60 F254 aluminium sheets (Merck ref. 1.05554, Millipore^®^) and visualized with UV light (254 nm).

^1^H NMR (400.1 MHz) and ^13^C NMR (100.6 MHz) were recorded on a Bruker Avance 400 spectrometer; chemical shifts were expressed as δ values and referenced to the residual solvent peak (DMSO-*d*_*6*_, δ_H_ = 2.50, δ_C_ = 39.5) and coupling constants were reported in units of Hertz (Hz).

The signals of ^1^H and ^13^C spectra were unambiguously assigned using 2D NMR techniques (COSY, HMQC, HMBC and NOESY) – see Supplementary Figures S1–S4 for ^1^H and ^13^C NMR spectra in Supplementary Material. FTIR spectra were obtained using a Nicolet 6700 (Thermo Electron Corp. Madison. WI) in KBr pellets and only the diagnostic absorption bands were reported in cm^−1^. HR-ESI-MS spectra were acquired in the positive mode on FT-ICR-MS Solarix XR Bruker Daltonics, 7 Tesla at the FCUL node of the Portuguese Mass Spectrometry Network.

The purity of the compounds (>98.1%) was evaluated by GC-MS analysis using a Shimadzu^®^ GCMS-QP2010 Plus, coupled with an autosampler AOC-20s, an automatic injector AOC-20i and a Teknokroma^®^ Sapiens-5MS capillary column (30 m × 0.25 mm × 0.25 µm; dimethyl/diphenylpolysiloxane 95%:5%), and operating with an EI source. The carrier gas was nitrogen with a flow of 1 ml/min. 1 μl of each sample was directly injected *via* the autosampler into a split/splitless programmable-temperature mode (10°C/min until 200°C, then held at this temperature for 2 min, raised to 300°C at 10°C/min and maintained for 15 min at this final temperature); the mass spectrometer transfer line was set at 250°C.

### Synthesis

INH-C_10_, N33 and N34 were obtained as fully described in reference (Martins et al., 2014). N33red and N34red were obtained by reduction of the parent Schiff base. Sodium cyanoborohydride (2 equiv.) was added portion wise to a solution of N33 or N34 in hot methanol. The reaction mixture was acidified with methanolic HCl 5M until pH 3; at that point the solution color changed to dark yellow. The reaction was kept under reflux and monitored by TLC until the starting material was consumed. The solvent was then evaporated under reduced pressure, giving an orange-yellow oil. This residue was dissolved in water and basified to pH > 7 by addition of a few drops of NaOH 6 M, to break up the possible cyanoborate adducts. The solution was then washed with brine and extracted several times with dichloromethane, the combined organic extracts were dried with MgSO_4_ and the solvent evaporated under reduced pressure affording crude yellowish solids. Pure N33red and N34red were obtained after column chromatography with Et_2_O/MeOH 96.5:3.5 and by recrystallization from CH_2_Cl_2_/petroleum ether 2:3, respectively (adapted from (Borch et al., 1971)). Both N33red and N34red are very stable in aqueous solution (PBS with 5% DMSO) for at least 48 h (Supplementary Figure S5).

Methyl 4-((2-isonicotinoylhydrazinyl)methyl)benzoate (N33red) Amorphous white powder η= 62.6% IR (KBr): ν (cm^−1^) = 3,283 (N-H amine), 3,238 (N-H amide), 3,049 (Ar C-H), 1718 (C=O ester), 1,667 (C=O amide), 1,280 (C-O aryl ester)*;* ^1^H NMR (400 MHz, DMSO-*d*_*6*_): δ (ppm) = 10.30 (d, 1H, *J* = 4.8 Hz, C=ONH), 8.69 (AA′ part of a AA′BB′ system, 2H, *J* = 4.6, 1.7 Hz, H-2/H-6), 7.92 (AA′ part of a AA′BB′ system, 2H, *J* = 7.9 Hz, H-3’/H-5′), 7.66 (BB″ part of a AA′BB′ system, 2H *J* = 4.6; 1.8 Hz, H-3/H-5), 7.53 (BB″ part of a AA′BB′ system, 2H, *J* = 7.9 Hz, H-2′/H-6′), 5.74 (m, 1H, NHCH_2_), 4.08 (d, 2H, *J* = 4.1 Hz, CH_2_), 3.84 (s, 3H,CH_3_); ^13^C NMR (100.6 MHz, DMSO-*d*_*6*_): δ (ppm) = 166.15 (OC= O), 163.92 (NHC= O), 150.19 (C-2/C-6), 144.24 (C-1′), 140.11 (C-4), 129.00 (C-3′/C-5′), 128.74 (C-2′/C-6′), 128.31 (C-4′), 121.04 (C-3/C-5), 53.95 (CH_2_), 52.02 (CH_3_). HR-ESI-MS: 286.11903 *m/z* [M+H]^+^ (calcd. for C_15_H_15_N_3_O_3_ +H, 286.11862)

*N*'-(4-phenoxybenzyl)isonicotinohydrazide (N34red) Amorphous white powder η= 50.6%, recrystallization from CH_2_Cl_2_: petroleum ether (2:3); IR (KBr): ν (cm^−1^) = 3,308 (N-H amine), 3,276 (N-H amide), 3,039 (Ar C-H), 1,640 (C=O amide), 1,247 (C-O aryl ether)*;* ^1^H NMR (400 MHz, DMSO-*d*_*6*_): δ (ppm) = 10.32 (d, 1H, *J* = 3.6 Hz, C=ONH), 8.70 (AA′ part of a AA’BB’ system, 2H, *J* = 4.6 Hz, H-2/H-6), 7.69 (BB″ part of a AA’BB’ system, 2H, *J* = 4.7 Hz, H-3/H-5), 7.38 (m, 4H, H-2’/H-6′, H-3″/H-5″), 7.12 (t, 1H, *J* = 7.1 Hz, H-4″), 6.97 (m, 4H, H-3’/H-5′, H-2″/H-6″), 5.55 (s_b_, 1H, *J* = 4.2 Hz, NHCH_2_), 3.97 (s, 2H, CH_2_); ^13^C NMR (100.6 MHz, DMSO-*d*_*6*_): δ (ppm) = 163.77 (NHC = O), 156.83 (COC), 155.63 (COC), 150.19 (C-2/C-6), 140.17 (C-4), 133.48 (C-1′), 130.34 (C-2′/C-6′), 129.97 (C-3″/C-5″), 123.28 (C-4″), 121.04 (C-3/C-5), 118.46 (C-2″/C-6″), 118.39 (C-3′/C-5′), 53.86 (CH_2_). HR-ESI-MS: 320.13929 *m/z* [M+H]^+^ (calcd. for C_19_H_17_N_3_O_2_+H, 320.13935).

### Lipophilicity Measurements

Lipophilicity was evaluated as log P_o/w_, the octanol-water partition coefficient obtained by the shake-flask method (Ràfols et al., 2012; Martins et al., 2014), and as log K_p(NaDS/w)_ the partition coefficient towards sodium dodecyl sulfate (NaDS) anionic micelles obtained by differential spectrophotometry for INH, INH-C_10_, N33 and N34 in previous works (Ràfols et al., 2012; Martins et al., 2014; Santos et al., 2020). For the new compounds, N33red and N34red, a preliminary assessment of their log P_o/w_ was achieved in this work, on the basis of the same general procedure already reported (Ràfols et al., 2012; Martins et al., 2014). For both compounds, samples were dissolved in water-saturated octanol at pH 7.5 (KH_2_PO_4_/NaOH) to guarantee negligible ionization. The partition to octanol-saturated aqueous buffer was evaluated, at room temperature, using a volume ratio, r_o/w_, of 5/30 and 10/30 for N33red and 2.5/40, 5/40 for N34red, after sonication of the solutions and partition on a mechanical tumbler. Equilibrium concentrations were determined by UV-Vis spectrophotometry between 200 and 500 nm after phase separation.

### HepG2 Cell Viability Assay

HepG2 cells were plated on 384-well tissue culture treated polystyrene plates at 2000 cells in 25 μl of HepG2 maintenance medium per well. After an overnight incubation at 37°C, the cells were dosed with test compounds in dimethyl sulfoxide (DMSO) and controls over wide ranges of concentrations (Table 1) and incubated for 72 h at 37°C. DMSO was at 0.5% in the final solution. The aqueous solubility of the compounds limited the range of concentrations tested. Cell viability was measured using the Promega CellTiter 96 Non-Radioactive Cell Proliferation Assay (MTT) kit by adding the Dye Solution to each well and incubating for 3 h at 37°C. After incubation, the Solubilization Solution/Stop Mix was added to each well. Plates were incubated at 37°C for 1 h, mixed on a plate shaker for 10 min and then absorbance was read at 570 nm. Cell viability was determined by comparison against control cells in the presence of DMSO only and was measured in three independent replicates for each compound concentration. The IC_50_ was calculated with GraphPad Prism using a Sigmoidal Dose-response (variable slope) algorithm. Chlorpromazine, a drug with a well-known and characterized hepatotoxicity (Frötschl et al., 2005; Gandhi et al., 2013; Morgan et al., 2019), was used as the positive control.

**TABLE 1:** Abbreviation (ID), systematic name, concentrations in HSA-binding and cytotoxicity studies and purity of the compounds used in this work.

ID	Systematic name	Concentrations studied (µM)		Purity (GC-MS)
		HSA-binding	Cytotoxicity	
INH	isonicotinoylhydrazide	0, 62.5, 125, 375, 625, 750, 1,000, 1,250, 1,750, 2,250, 4,000 and 6,000	0.063, 0.2, 0.63, 2, 6.3, 20, 63.3 and 200	99.4
INH-C_10_	*N*′-decanoylisonicotinoylhydrazide	0, 2.6, 5.2, 10.4, 20.8, 26.0, 31.2, 62.5, 125 and 200	0.0079, 0.025, 0.079, 0.25, 0.79, 2.5, 7.9 and 25	98.1
N33	methyl (*E*)-4-((2-isonicotinoylhydrazineylidene)methyl)benzoate	0, 2.6, 5.2, 10.4, 20.8, 26.0, 31.2, 62.5, 125 and 200	0.0079, 0.025, 0.079, 0.25, 0.79, 2.5, 7.9 and 25	99.5
N34	(*E*)-*N*'-(4-phenoxybenzylidene)isonicotinohydrazide	0, 2.6, 5.2, 10.4, 20.8, 26.0, 31.2, 62.5, 125 and 200	0.063, 0.2, 0.63, 2, 6.3, 20, 63.3 and 200	98.5
N33red	methyl 4-((2-isonicotinoylhydrazinyl)methyl)benzoate	0, 2.6, 5.2, 10.4, 20.8, 26.0, 31.2, 62.5, 125, 200, 300 and 400	0.063, 0.2, 0.63, 2, 6.3, 20, 63.3 and 200	98.1
N34red	*N*'-(4-phenoxybenzyl)isonicotinohydrazide	0, 2.6, 5.2, 10.4, 20.8, 26.0, 31.2, 62.5, 125, 200, 300 and 400	0.0158, 0.05, 0.158, 0.5, 1.58, 5, 15.8 and 50	98.5

### Determination of Minimum Inhibitory Concentrations

The isoniazid derivatives were screened *in vitro* for its activity against *M. tuberculosis* by broth macrodilution using the BACTEC MGIT 960 system and the growth monitored, at 37°C, with the Epicenter V5.80A software (Becton Dickinson, Diagnostic Systems, Sparks, MD, United States) as previously described (Springer et al., 2009; Martins et al., 2014). The MGIT tubes were inoculated with 0.8 ml of OADC (oleic acid, albumin, dextrose, catalase; Becton and Dickinson), 0.1 ml of the compound at the pretended concentration and 0.5 ml of the strain suspension. The compounds were tested from 0.03125 to 128 µM. At the time of testing, two-fold serial dilutions were prepared to achieve the desired concentrations. When positive growth was observed in the drug containing tube (GU ≥ 100) before the positivity of the proportional growth control (containing a 1/100 dilution of the suspension of the strain), this indicates that more than 1% of the population was able to grow in the presence of the concentration of the anti-TB drug and, as per the proportion testing method definition recommended by WHO, the strain is considered resistant at the corresponding drug concentration (Canetti et al., 1963). Thus, the MIC was the lowest concentration with GU < 100 when the drug-free 1/100 proportional control tube reached the positivity threshold of 400 GU. An absolute growth control (undiluted) was included in every assay to monitor the normal growth of each strain. The assays were performed in triplicate (biological replicates) and the final value was given as the result of two concordant values (Machado et al., 2016).

The *M. tuberculosis* H37Rv ATCC27294^T^ reference strain was obtained from the American Type Culture Collection (Virginia, United States). The *M. tuberculosis* clinical strain, monorresistant to isoniazid due to the presence of the mutation S315T in the *katG* gene (Machado et al., 2018, Machado et al., 2016), was isolated in 2003 from a Portuguese patient as part of the routine mycobacteriology laboratory services provided by Universidade NOVA de Lisboa (Lisboa, Portugal) to the local hospitals. Informed consent was not required for this study as it involves only anonymized bacterial isolates.

### Sample Preparation for Spectroscopic Measurements With Albumin

HSA stock solutions were prepared by gently dissolving the protein in PBS (Matos et al., 2013). The concentration of the HSA stock solutions was 10.4 µM as determined by spectrophotometry using the molar absorption coefficient reported by Pace *et al.* (*ε*_280 nm_ = 35,219 M^−1^ cm^−1^) (Pace et al., 1995).

INH and INH derivatives stock solutions were prepared in DMSO. From the stock solutions, appropriate dilutions were prepared so that each compound was added to HSA in PBS at different concentrations ensuring that DMSO was at a fixed concentration of 5% (v/v) in the final solution. HSA concentration in the final solution was 5.2 µM. Due to limitations imposed by the solubility of each compound, the range of concentrations assayed varied for each compound as shown in Table 1. Prior to measurements, the samples were incubated at 37.0 ± 0.5°C for 24 h. Other incubation times were tested as well – 48, 72 and 96 h – and since no changes in the data obtained were observed the incubation time was set to 24 h. Three or more independent replicates were prepared and analyzed for each system.

For the competitive binding assays with warfarin the same preparation protocol was carried out, except that the solution also contained warfarin in equimolar proportion to HSA (5.2 µM) equilibrated prior to incubation with the compound. Warfarin stock solution was also prepared with DMSO.

### Spectroscopic Measurements and Data Analysis

Fluorescence measurements were performed with a Fluorolog 3.22 spectrofluorimeter (Horiba Jobin Yvon, Villeneuve D'ascq, France) at 24.0 ± 0.5°C in a sample compartment with temperature-control. Quartz Suprasil^®^ cuvettes with 1 × 0.4 cm path length were used to gather the spectroscopic data.

For steady-state fluorescence intensity measurements, the excitation wavelength was set to 295 nm and emission was collected in the range 310–550 nm. The bandwidth was 4 nm in both excitation and emission paths. The fluorescence intensity was corrected for the absorption and emission inner filter effects using the absorption spectra recorded for each sample (Lakowicz, 2006). Electronic absorption spectra were recorded at room temperature with a Jasco V-560 spectrophotometer (Hiroshima, Japan) in the range from 250 to 500 nm.

Time-resolved fluorescence measurements were carried out by the single photon counting technique, using a nanoLED N-280 (Horiba Jobin Yvon, Villeneuve D'ascq, France) for the excitation of the protein. The emission wavelength was set to 350 nm in order to avoid the contribution from tyrosine residues and collect only the photons emitted by the tryptophane residue ^214^Trp (Starosta et al., 2020). The bandwidth was 11 nm and a timescale of 55ps/channel (1,024 channels) was employed. Ludox^®^ was the scattering agent used to obtain the instrumental response function. The experimental fluorescence intensity decays were analysed with the software TRFA^®^ version 1.4 (Minsk, Belarus). The fluorescence intensity decays, *I(t)*, were analysed by fitting a sum of exponentials, and the decay law was then obtained according to Eq. 1:

$$I\left(t\right)=\;\sum_{\text{i}=1}^{\text{n}}\alpha_{i}\exp\left(-\frac{t}{\tau_{i}}\right)$$ (1)

where *α*_i_ and *τ*_i_ are the normalized amplitude and lifetime of component *i*, respectively.

The changes in quantum yield due to processes affecting the fluorescence lifetime were assessed from the amplitude-weighted mean fluorescence lifetime, $\bar{\tau }$ , calculated using Eq. 2:

$$\bar{\tau }=\sum_{i=1}^{n}\alpha_{i}\tau_{i}$$ (2)

The criteria to ascertain the good quality of the fitting were the reduced χ^2^ value close to 1 and the random distributions of weighted residuals and residuals autocorrelation.

Regarding both steady-state and time-resolved measurements, an adequate blank was subtracted from each reading and at least three independent experiments were performed for each compound. The results are presented as mean ± standard deviation. Unless otherwise stated, statistical significance versus INH was determined by one-way ANOVA with Tukey’s *post-hoc test* (*, *p* < 0.05; **, *p* < 0.01; ***p < 0.001) (Mishra et al., 2019).

## Results and Discussion

Four different and complementary *in vitro* approaches, deemed to be suitable considering the current stage of development of this project, were employed to assess the potential *in vivo* application of the INH derivatives under study to treat MDR-TB.

### *In vitro* Evaluation of Hepatotoxicity

In early screening of potential drugs, it is important to assess their *in vitro* cytotoxicity to be able to justify further investments in their development. In fact, cell viability assays to evaluate drug toxicity are frequently employed during the process of drug development (Riss et al., 2013; Jakštys et al., 2015; Niepel et al., 2019). In general terms, drugs can exert their toxicity through on-target pharmacology, *i.e.*, by an exacerbated action on the primary pharmacological target, or through off-target toxicity, which is a common mode of toxicity for small-molecule drugs (and/or their metabolites) as a result of their pharmacological promiscuity and/or their unintended chemical reactivity with biomolecules (Muller and Milton, 2012).

Many drugs in clinical use exhibit, at some point, some degree of hepatotoxicity (Devarbhavi, 2012). To evaluate the *in vitro* toxicity of this series of INH derivatives, the viability of HepG2 cells, a human liver cancer cell line, was determined by the MTT tetrazolium reduction assay (Riss et al., 2013), a colorimetric assay to assess cell metabolic activity, and the IC_50_ value, *i.e*., the concentration for 50% reduction in cell viability, was obtained for each compound (Supplementary Figure S6). The IC_50_ value for the positive control, chlorpromazine, a drug mainly used in the treatment of psychotic disorders, was 13.9 µM, which is consistent with a previous assessment of IC_50_—34.7 µM against the same cell line, but a shorter incubation time (24 h instead of 72 h) (Wang et al., 2002). The hepatoxicity of INH is well described (Girling, 1978; Wu and Cederbaum, 1996; Huang et al., 2003; Bhadauria et al., 2007; Singh et al., 2011) and is much lower than that of chlorpromazine. From studies using HepG2 cells it has been suggested that the main mechanisms by which INH may exert toxic effects is by inducing oxidative stress, mitochondria dysfunction and apoptosis (Bhadauria et al., 2007).

Here, we intended to assess the toxicity of these INH derivatives as a pre-screening approach to evaluate the suitability of these compounds to be considered in the treatment of TB. The results obtained in cell viability assays (Supplementary Figure S6, Table 2 (in µM) and Supplementary Table S1 (in μg ml^−1^)) suggest that most compounds tested are not toxic within the concentration ranges studied. The parental drug INH as well as compounds N34 and N33red did not significantly affect HepG2 cell viability up to 200 µM. Due to limitations imposed by the aqueous solubility of the compounds, the effects of INH-C_10_ and N33 were only tested up to 25 µM. At this concentration INH-C_10_ only marginally affected cell viability, whereas N33 had no significant impact (Supplementary Figure S6). N34red was the only INH derivative for which it was possible to determine an IC_50_ value, *i.e*., 48.5 µM. All the IC_50_ values obtained show that INH and the studied derivatives are less toxic than drugs currently in clinical use. Rifampin, an antibiotic employed for the treatment of mycobacterial infections, has an IC_50_ value of 25.5 µM (Vahdati-Mashhadian et al., 2013). More importantly, all of these INH derivatives seem to be safer than bedaquiline, which has an IC_50_ of 17.4 µM (Lupien et al., 2018) and half of them are certainly better, in terms of cytotoxicity, than delamanid, which presents an IC_50_ of 98.9 µM (Lupien et al., 2018). The IC_50_ presented for bedaquiline and delamanid were obtained in the same conditions, *i.e.*, against the same cell line, HepG2, and the same incubation time (72 h) (Lupien et al., 2018).

**TABLE 2:** IC_50_ values of the compounds studied in this work against HepG2 cells (including the positive control, chlorpromazine), MIC values against *wt* (MIC (*wt*)) and *katG* (S315T) (MIC (*katG* S315T)) strains of *Mtb* and selectivity index computed with the activity against *wt* (SI (*wt*)) and *katG* S135T (SI (*katG* S315T)).

Compound	IC_50_/µM	MIC (*wt*)/µM	MIC (*katG* S315T)/µM	SI (*wt*)	SI (*katG* S315T)
INH	>200	0.29^a^	43.8^a^	>690	>4.6
INH-C_10_	>25	0.38^a^	6.9^a^	>66	>3.6
N33	>25	1.06^a^	21.2^a^	>24	>1.2
N34	>200	0.95^a^	18.9^a^	>210	>10.5
N33red	>200	2.0	>128	>100	n.d.
N34red	48.5	1.0	>128	48.5	<0.4
Chlorpromazine	13.9				

a Retrieved from reference (Martins et al., 2014).

The selectivity index, SI, which depends on the toxicity of the compound, is also an important parameter when considering the pharmaceutical application of new drugs. In a previous work we determined the activity of a series of INH derivatives against the wild type strain of *Mtb* (H37Rv) and the *katG* (S315T) mutant strain that lies as the most frequent and important cause of INH resistance worldwide (Martins et al., 2014). The minimum inhibitory concentrations (MIC), defined as the lowest concentration necessary to inhibit 99% of the bacterial population, established in (Martins et al., 2014) using the BACTEC™ MGIT™ 960 system (BACTEC 960) and the Epicenter V5.53A software, together with the IC_50_ values determined in the present work against HepG2 cells, can be used to estimate the *in vitro* SI according to Eq. 3:

$$SI\;=\frac{IC_{50}}{MIC}$$ (3)

In Table 2, the SIs considering the activity against both the wild type and mutant strains are presented. The data gathered so far indicates that, by comparison with the other studied compounds, INH presents the best performance against the *wt Mtb*; however, when considering the mutant strain *katG* (S315T), the SI of N34 makes it a very promising compound. In addition, two other INH derivatives also present higher activity against this strain when compared with INH, which in the case of INH-C_10_ corresponds to a 6-fold gain in efficacy. At this point, the solubility limit of this compound in buffer with 0.5% DMSO prevents us to elaborate further on the SI for this compound. Yet, the *in vitro* efficiency of a drug can greatly differ from that *in vivo* (Braggio et al., 2010). Drug efficiency is defined as the fraction of the dose administered that effectively reaches the site of action (Braggio et al., 2010). While *in vitro* this parameter is expected to be near 1, *in vivo* it greatly depends on the ADME processes, which can be profoundly influenced by the plasma protein binding ability of the drugs.

### Albumin Binding Studies and Correlations With Compound Lipophilicity

It is common understanding that the binding to plasma proteins can not only modulate the pharmacokinetics of a drug, but also lower its toxicity (Schmidt et al., 2010; Wanat, 2020). Thus, in addition to the SI, plasma protein binding is also an important aspect to take into consideration in the evaluation of potential drugs. In fact, many of the highly prescribed drugs bind to plasma proteins in a percentage greater than 98% (Smith et al., 2010). This constitutes the rationale behind the evaluation of HSA interactions with INH and its derivatives, since HSA is the most abundant protein in the plasma, and, as already referred, also the most important non-specific drug carrier.

**FIGURE 2:**
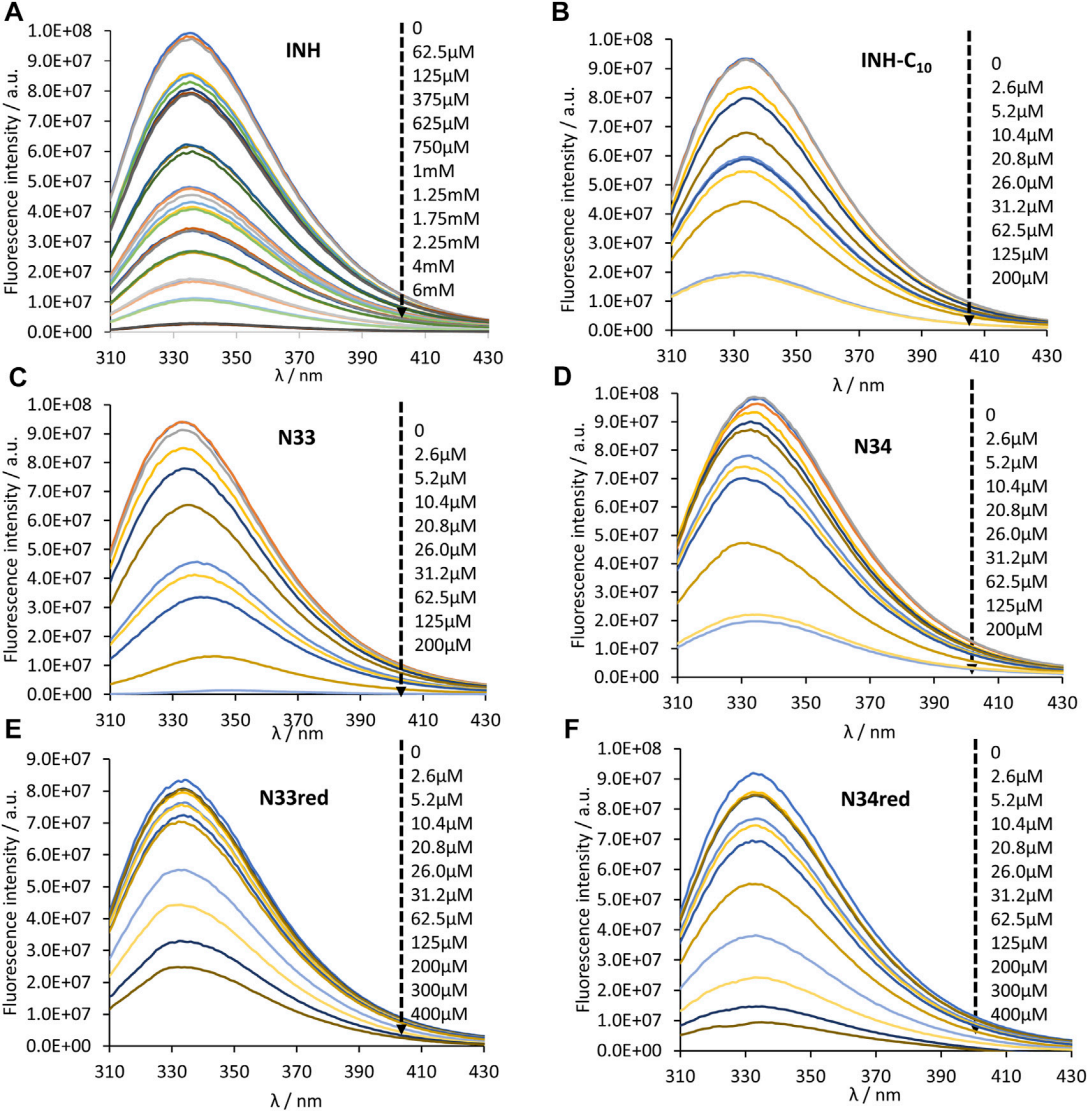
Steady-state emission spectra of HSA ^214^Trp in the absence and presence of **(A)** INH, **(B)** INH-C_10_, **(C)** N33, **(D)** N34, **(E)** N33red and **(F)** N34red. ^214^Trp fluorescence quenching (λ_ex_ = 295 nm) is observed upon compound binding (black dashed arrow indicates the direction of increasing compound concentration). The conditions were as follows: [HSA] = 5.2 µM, kept constant; samples prepared in PBS, pH 7.4; 24 h incubation at (37.0 ± 0.5)°C; measurements at room temperature, (24.0 ± 0.5)°C.

**FIGURE 3:**
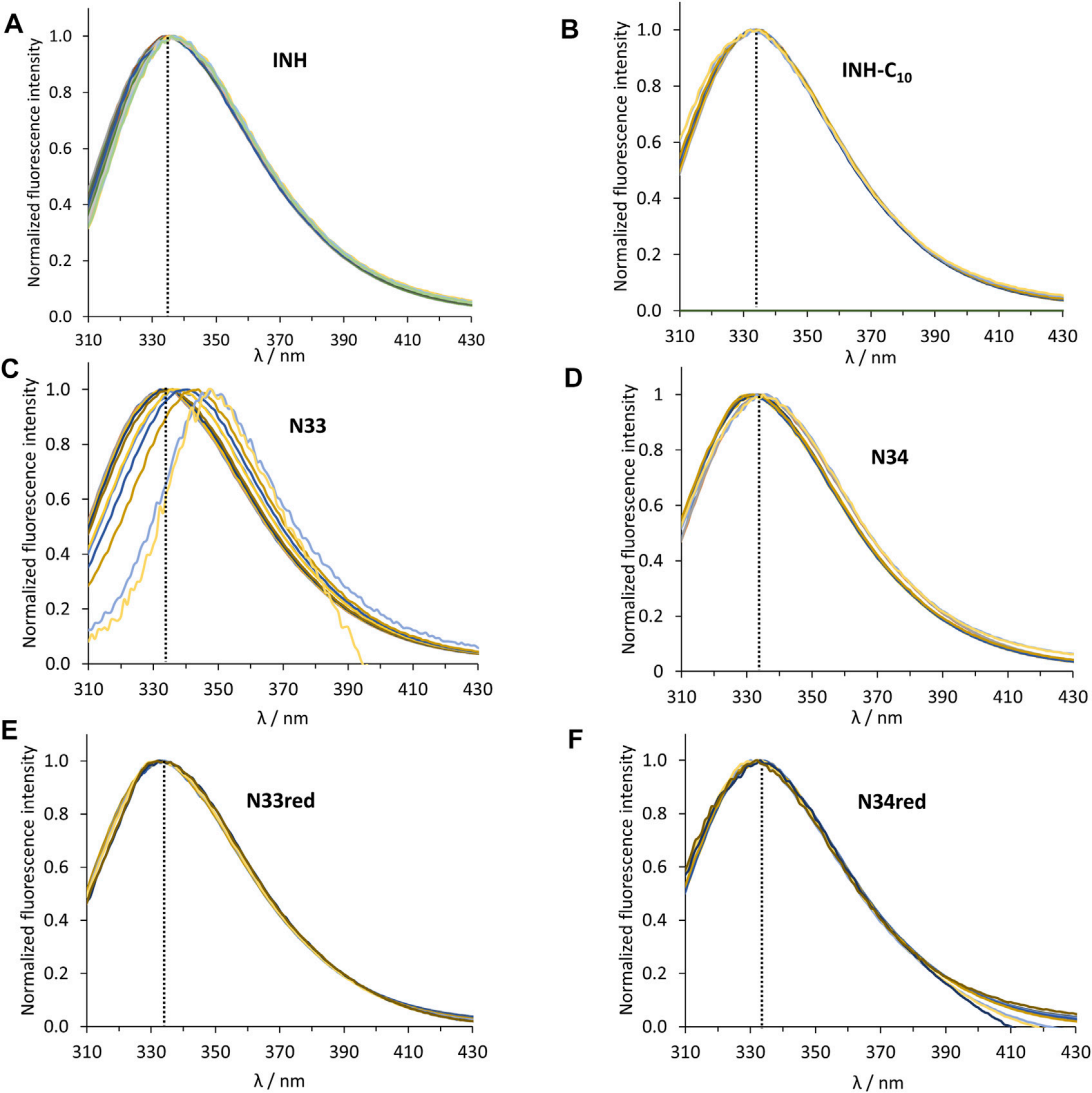
Normalized steady-state emission spectra of HSA ^214^Trp in the absence and presence of **(A)** INH, **(B)** INH-C_10_, **(C)** N33, **(D)** N34, **(E)** N33red and **(F)** N34red. The dashed black line indicates the maximum emission of HSA in the absence of any compound (λ = 334 nm). The conditions were as follows: [HSA] = 5.2 µM, kept constant; λ_ex_ = 295 nm; samples prepared in PBS, pH 7.4; 24 h incubation at (37.0 ± 0.5)°C; measurements at room temperature, (24.0 ± 0.5)°C.

HSA contains 18 tyrosine residues and a single tryptophane residue, ^214^Trp, located in subdomain IIA. The interaction between INH or its derivatives and HSA can be studied by fluorescence spectroscopy by selective excitation of ^214^Trp at 295 nm and emission collection at 340 nm or longer wavelengths (Luís et al., 2014; Teale and Weber, 1957). Although ^214^Trp is in the vicinity of Sudlow’s binding site I, its photophysics also reports changes occurring in binding site II due to its high sensitivity to changes in the environment either as a result of drug binding or of structural alterations of the protein (Luís et al., 2014). The emission spectra of HSA in the presence of increasing concentrations of each compound can be found in Figure 2. The maximum emission intensity of HSA in the absence of any compound was observed at 334 nm in agreement with previous observations (Demoro et al., 2013; Luís et al., 2014), indicating that ^214^Trp is shielded from the aqueous solvent, since tryptophane has a typical emission maximum close to 350 nm in water (Teale and Weber, 1957). As a general acknowledgment, with the increment in the concentration of the compounds there is a pronounced decrease in the emission intensity of HSA, thus all compounds have the ability to quench the fluorescence of ^214^Trp. Moreover, in the normalized emission spectra, it can be observed that INH, INH-C_10_ N33red and N34red did not promote any spectral shifts (Figures 3A,B,E,F), whereas N33 induced a red shift (Figure 3C) and N34 may have caused a small blue shift (Figure 3D). Therefore, while INH, INH-C_10,_ N33red and N34red binding do not seem to induce structural alterations in the protein that change the exposure degree of ^214^Trp, the same is not verified for N33 and possibly N34. The red shift in the maximum emission upon the binding of N33 points to a structural change that seems to leave ^214^Trp exposed to a more polar environment, suggesting a greater water accessibility in the vicinity of that amino acid residue. In fact, for the highest concentration of N33, the maximum emission is shifted to ∼350 nm, which is close to the maximum emission of ^214^Trp in water. On the other hand, the blue shift effect of N34 is much smaller (ca. 5 nm).

**FIGURE 4:**
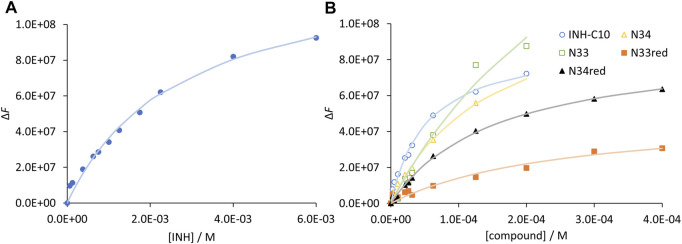
Compound binding to HSA as assessed by steady-state fluorescence spectroscopy. Variation of fluorescence intensity for increasing concentration of **(A)** INH and **(B)** INH derivatives. The dots correspond to experimental data and the lines correspond to the non-linear fit of Eq. 5. Analysis is based on the fluorescence intensity at λ_em_ = 340 nm (λ_ex_ = 295 nm), under the following conditions: [HSA] = 5.2 µM; samples prepared in PBS, pH 7.4; 24 h incubation at (37.0 ± 0.5) °C; measurements at room temperature, (24.0 ± 0.5)°C. INH was represented separately due to the very different concentration range assayed.

For further analysis, the fluorescence intensity values were taken at excitation and emission wavelengths of λ_ex_ = 295 nm and λ_em_ = 340 nm and corrected for the inner filter effects, yielding a corrected fluorescence intensity value, *F*, for each sample. The variation of such fluorescence intensity (Δ*F*) between the samples containing only HSA (*F*_0_) and HSA + compound (*F*) can be calculated for each concentration of the compound to estimate the dissociation constant (*K*_d_) for the equilibrium HSA + compound. As detailed in (Luís et al., 2014), plotting Δ*F* as a function of compound concentration ([*C*]), Eq. 4:

$$\Delta F=\frac{\left[C\right]}{K_{d}+\left[C\right]}\Delta F_{max}$$ (4)

enables the computation of *K*_d_ values and of the maximum variation in fluorescence intensity (Δ*F*_max_) for unlimited compound concentration, by a non-linear fit with Eq. 4. In Figure 4 the graphical representation of Δ*F* as a function of [*C*] is shown for INH (Figure 4A) and all INH derivatives (Figure 4B) studied in this work, and *K*_d_ values for all the compounds are presented in Table 3.

**TABLE 3:** Dissociation constants of the compounds studied in this work for HSA retrieved from the variation in the steady-state fluorescence intensity (*K*_d_), from the variation in the fluorescence intensity corrected regarding the variation in fluorescence lifetime (*K′*_d_) and from the variation in $\bar{\tau }$ (*K″*_d_). The reverse of the constants computed from the slope in the Stern Volmer plot of fluorescence intensities corrected regarding the variation in fluorescence lifetime are also presented (1/*K*), as well as the dissociation constants of the compounds for the warfarin binding site in the presence of equimolar proportions of HSA and this competitor (*K*_dc_). Octanol-water partition coefficients (log *P*_o/w_). are also shown. All values result from the average of at least 3 independent experiments and are presented with the respective standard deviations (*SD*). Statistical significance versus INH: **, *p* < 0.01.

Compound	10^3^ (*K*_d_ ± *SD*)/M	10^3^ (*K′*_d_ ± *SD*)/M	10^3^ (1/(*K* ± *SD*))/M	10^3^ (*K′′*_d_ ± *SD*)/M	10^3^ (*K*_dc_ ± *SD*)/M	log *P*_o/w_ ± *SD*
INH	2.72 ± 0.04	2.72 ± 0.04	1.65 ± 0.06	-	4.55 ± 0.05	−0.85 ± 0.01^b^
INH-C_10_	0.046 ± 0.009**	0.05 ± 0.02**	0.07 ± 0.01**	0.120 ± 0.003	0.4 ± 0.2**	3.5 ± 0.2^b^
N33	0.16 ± 0.05**	0.2 ± 0.1**	0.12 ± 0.04**	0.08 ± 0.06	0.054 ± 0.004**	1.32 ± 0.06^c^
N34	0.13 ± 0.05**	0.11 ± 0.05**	0.07 ± 0.03**	0.52 ± 0.02^a^	0.128 ± 0.005**	3.7 ± 0.1^c^
N33red	0.6 ± 0.2**	0.6 ± 0.2**	0.56 ± 0.05**	-	0.9 ± 0.3**	1.33 ± 0.04
N34red	0.15 ± 0.03**	0.14 ± 0.04**	0.090 ± 0.001**	0.73 ± 0.06^a^	0.34 ± 0.02**	2.6 ± 0.2

a K values correspond to the slopes obtained from the Stern Volmer plot using the amplitude-weighted mean fluorescence lifetimes.

b Retrieved from reference (Ràfols et al., 2012).

c Retrieved from reference (Martins et al., 2014).

Regarding the data in Figure 4 the emission intensity at 340 nm was used instead of that at 334 nm to avoid water Raman scattering and improve the signal-to-background ratio, while maintaining very good sensitivity (close to maximum) (Demoro et al., 2013; Matos et al., 2013). The *K*_d_ of INH in the range of 10^−3^M (Table 3), as determined by us, represents a weak interaction as compared to other drugs, which typically display *K*_d_ in the range of 10^−4^–10^−6^M (Sowell et al., 2001; Chen and Hage, 2006; Kanakis et al., 2006; Kim and Wainer, 2008; Mallik et al., 2008; Joseph et al., 2010; Joseph et al., 2011; Matsuda et al., 2011; Yang et al., 2014). A *K*_d_ value of 3.9 ± 0.4 × 10^−4^ M has been previously reported for INH (Ascenzi et al., 2011). However, it is not mentioned in that study if the fluorescence intensity was corrected for inner filter effects. In fact, if we use the uncorrected fluorescence intensity data to calculate INH *K*_d_, we obtain a value of 6.5 × 10^−4^ M (Supplementary Figure S7).

**FIGURE 5:**
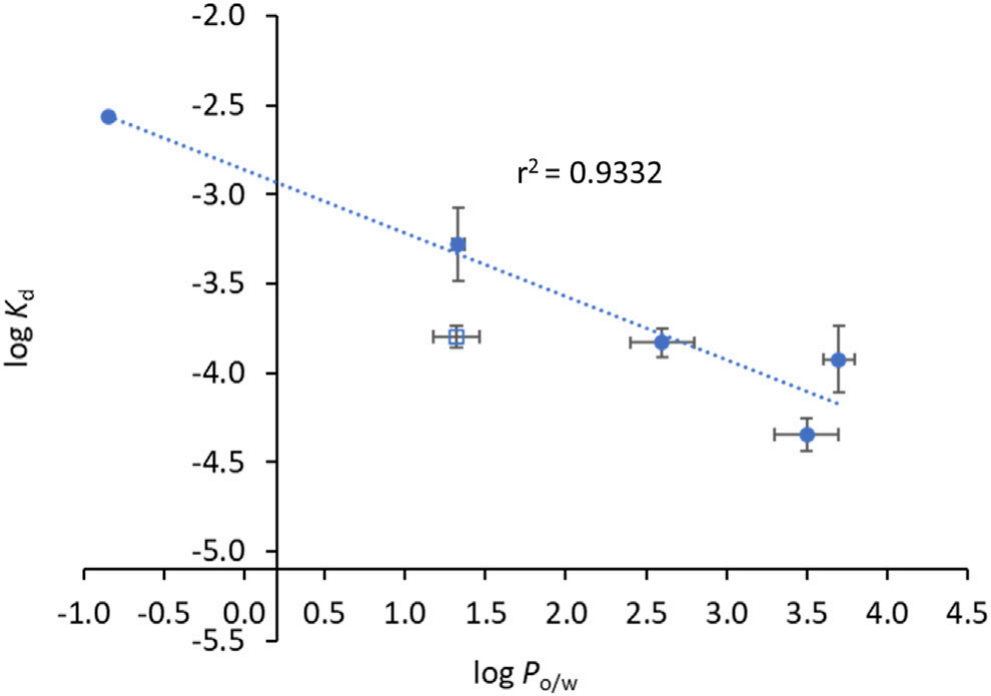
Compounds’ binding to HSA is correlated with their lipophilicity. The dots correspond to experimental data and the line corresponds to a linear fit to the data points. log P_*o/w*_ and log *K*_d_ values can be found in Table 3. Data points used for the linear fit included INH, INH-C_10_, N34, N33red and N34red. The rational as to why N33, open square, is not used for the linear fit is given in the text. The relationship between log *K*_d_ and log *P*_o/w_ is given by log *K*_d_ = -(0.35 ± 0.05) log *P*_o/w_ – (2.86 ± 0.14).

Notably, all INH derivatives exhibit a more favourable interaction with HSA as compared to INH (Table 3). INH-C_10_, which was the compound with the strongest antimicrobial activity against the *katG* S315T mutant strain of *Mtb*, is also the one with more affinity for HSA. Compound N34, the one that displayed the most promising SI regarding the activity against the mutant strain, also presents a *K*_d_ value that is one order of magnitude lower than that of INH, meaning that binding is one order of magnitude stronger. The *K*_d_ determined for the INH derivatives lay within the range of many clinically used drugs, 10^−4^–10^−6^, (Smith et al., 2010) which is another encouraging result regarding future efforts in the development of alternative therapies to treat MDR-TB based on the chemical tailoring of INH. The binding of the compounds tested to HSA displays a good correlation with their lipophilicity, as expressed by their octanol-water partition coefficients (Table 3), which is clearly shown in the plot of log *K*_d_ *vs*. log *P*_o/w_ (Figure 5). N33 deviates from the linear trend and was not considered in the linear regression. Such deviation probably occurs because this compound induces a structural change in the protein that leaves ^214^Trp exposed to water as suggested by the strong emission red shift (Figure 3C), unlike all other compounds. The correlation found between log *K*_d_ and log *P*_o/w_ follows the typical trend for compound-plasma protein binding, *i.e.*, as compounds are more lipophilic the more significant becomes their binding to plasma proteins (Croom, 2012). As mentioned, the range of *K*_d_ of the derivatives is equivalent to other drugs. Higher *K*_d_ would result in an increased free fraction of the drug, whereas much lower *K*_d_ hinders the release of the compound by HSA. The same rational can be applied to lipophilicity, since a low lipophilicity implies a low partition towards lipid membranes, thus a limited ability to reach the target inside *Mtb*. On the other hand, a very high lipophilicity corresponds to a very high affinity for lipid membranes, which may result in a compound being trapped in the membrane once it penetrates it. We have previously shown that the lipophilicity of INH-C_10_ (Santos et al., 2020) is important for the accumulation of this compound in lipid membranes with a concomitant increase in bioavailability inside the cell (Vila-Viçosa et al., 2017).

**FIGURE 6:**
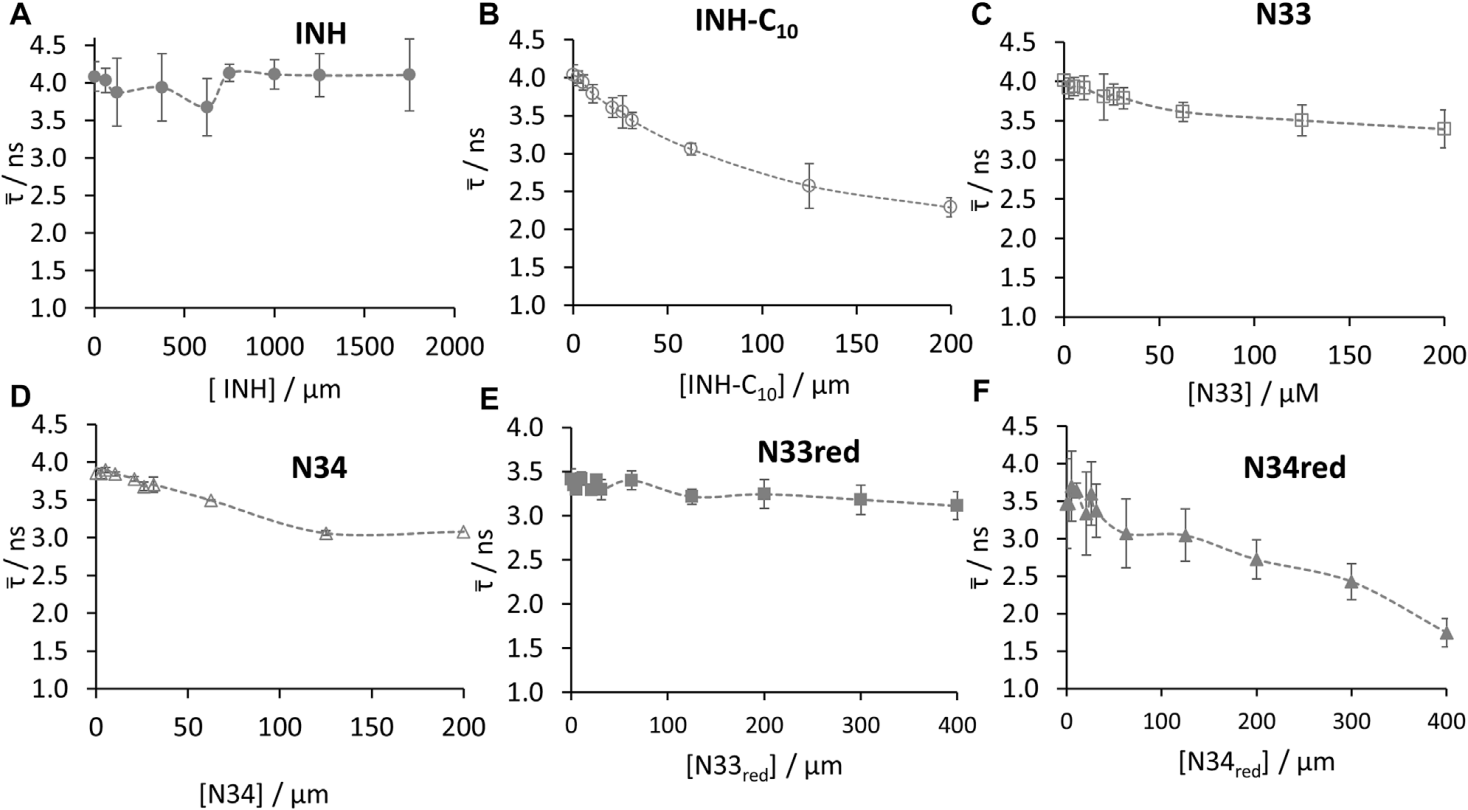
Amplitude-weighted mean fluorescence lifetime ( $\bar{\tau }$ ) (Eq. 3) of HSA ^214^Trp in the presence of increasing concentrations of **(A)** INH, **(B)** INH-C_10_, **(C)** N33, **(D)** N34, **(E)** N33red and **(F)** N34red (λ_ex_ = 279 nm; λ_em_ = 350 nm). Dashed line is merely a guide to the eye. Other experimental conditions: [HSA] = 5.2 µM, kept constant; λ_ex_ = 279 nm; λ_em_ = 350 nm; samples prepared in PBS, pH 7.4; 24 h incubation at (37.0 ± 0.5)°C; measurements at room temperature, (24.0 ± 0.5)°C.

**FIGURE 7:**
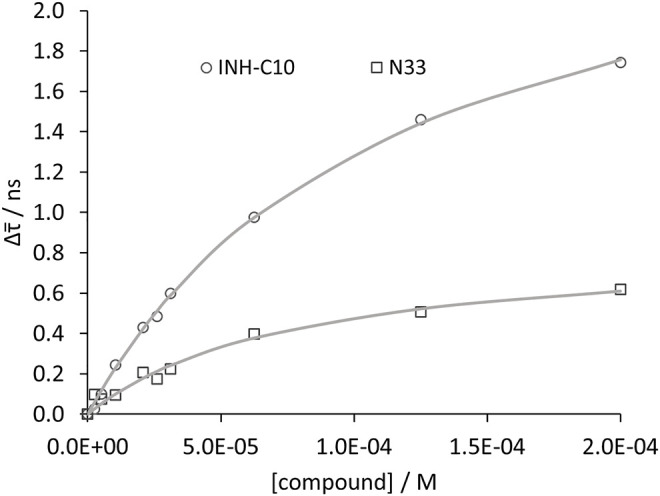
Compound binding to HSA as assessed by time resolved fluorescence spectroscopy. Variation of $\bar{\tau }$ with increasing concentrations of INH-C_10_ and N33. The dots correspond to experimental data and the lines correspond to the non-linear fit using Eq. 5. Analysis is based on the fluorescence intensity decay at λ_ex_ = 279 nm, λ_em_ = 350 nm. Other experimental conditions: [HSA] = 5.2 µM, kept constant; samples prepared in PBS, pH 7.4; 24 h incubation at (37.0 ± 0.5)°C; measurements at (24.0 ± 0.5)°C.

Regarding the fluorescence intensity decays of HSA ^214^Trp, the best fit was always obtained using three exponential terms. The normalized amplitude and lifetime of each component (*α*_1_ = 0.261, *α*_2_ = 0.428, *α*_3_ = 0.314, *τ*_1_ = 0.662 ns, *τ*_2_ = 3.443 ns, *τ*_3_ = 6.935 ns), that best described the fluorescence intensity decay of HSA alone are in good agreement with the ones previously reported (Luís et al., 2014; Starosta et al., 2020). As depicted in Figure 6 and Supplementary Figure S8 not all compounds have the same efficiency reducing the fluorescence lifetime of ^214^Trp. The invariance of the amplitude-weighted mean fluorescence lifetime ( $\bar{\tau }$ ) of ^214^Trp in the presence of increasing amounts of INH and N33red (Figures 6A,E) indicates that the decrease in steady-state fluorescence intensity is due to the association of the quencher with the fluorophore. On the contrary, for INH-C_10_, N33, N34 and N34red a decrease in the $\bar{\tau }$ of ^214^Trp as the concentration of compound increases was observed (Figures 6B–D,F). Performing a non-linear fit to the variation of $\bar{\tau }$ according to Eq. 4, where the steady-state fluorescence intensity is replaced by the amplitude-weighted mean fluorescence lifetime (Figure 7), it is possible to estimate the dissociation constants by time-resolved fluorescence measurements (*K*″_d_) which are also shown in Table 3. *K*″_d_ values reported for INH-C_10_ and N33 were obtained using a non-linear fit using Eq. 4. In the other two cases (N34 and N34red), the non-linear fit was not possible since the concentrations tested were not sufficiently high to define the binding curve plateau, and a linear fit to Eq. 5 (Stern-Volmer equation) was used instead. The Stern-Volmer relationship is given by

$$\frac{F_{0}}{F}=1+K\left[C\right]$$ (5)

where *F*_0_ and *F* are the fluorescence intensities in the absence and presence of a concentration of quencher compound, [*C*]. When used to analyse time-resolved fluorescence data, *F* is replaced by $\bar{\tau }$ . The *K*″_d_ values obtained for INH-C_10_ and N33 retrieved from either method are identical.

Whenever the decrease in $\bar{\tau }$ is smaller than the decrease in the steady-state fluorescence intensity, there are multiple quenching mechanisms responsible for a decrease in fluorescence quantum yield, one affecting the amplitude-weighted mean fluorescence lifetime and the fluorescence intensity alike, and another affecting only the fluorescence intensity (a static quenching mechanism). Therefore, for each data point, the decrease in fluorescence intensity that was not due to a decrease in $\bar{\tau }$ was calculated. These values were used to obtain yet another dissociation constant that corresponds to the binding process affecting only the steady-state fluorescence intensity, also shown in Table 3 (*K*’_d_). Upon this correction, the resulting *K*’_d_ are, in general, very close to the previously calculated *K*_d_, thus the variation in the fluorescence lifetime in most cases barely affected the dissociation constants estimated by steady-state fluorescence intensity. This is a consequence of the much larger relative decrease in fluorescence intensity when compared with the decrease in fluorescence lifetime.

**FIGURE 8:**
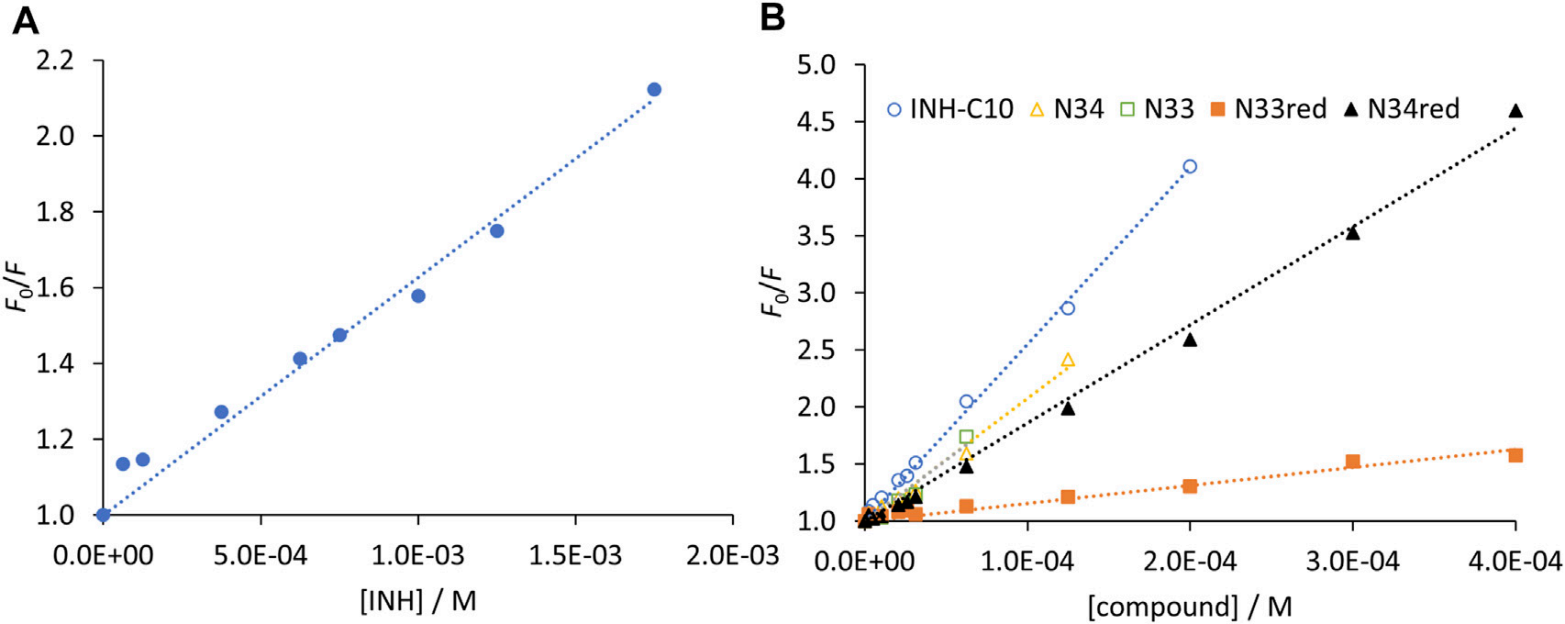
Stern-Volmer plots of HSA ^214^Trp obtained from the steady-state fluorescence intensity measurements after removing the contribution of the fluorescence lifetimes decrease for **(A)** INH and **(B)** INH derivatives. The dots correspond to experimental data and the lines correspond to the linear fit to Eq. 6. Analysis based on the fluorescence intensity at λ_em_ = 340 nm (λ_ex_ = 295 nm), under the following conditions: [HSA] = 5.2 µM; samples prepared in PBS, pH 7.4; 24 h incubation at (37.0 ± 0.5)°C; measurements at room temperature, (24.0 ± 0.5)°C. INH was represented separately due to the very different concentration range assayed.

The Stern–Volmer plots for HSA static quenching by the compounds, *i.e.*, the quenching obtained from the steady-state fluorescence intensity corrected for the decrease of the fluorescence lifetimes are shown in Figure 8.

For a static quenching mechanism, *K* represents the association constant for the formation of non-fluorescent HSA-compound ground-state complex. Therefore, the reverse of K gives an estimate of the dissociation constant. By fitting a straight line to the data in Figure 8 forcing a unitary intercept, *i.e.*, assuming a Stern-Volmer-like behaviour, a reasonable correlation coefficient was obtained in all cases. It is worth noting that the reverse of the Stern-Volmer constants, 1/*K* (Table 3), yields values mostly identical to the *K′*_d_ determined by the non-linear fit according to Eq. 4.

**FIGURE 9:**
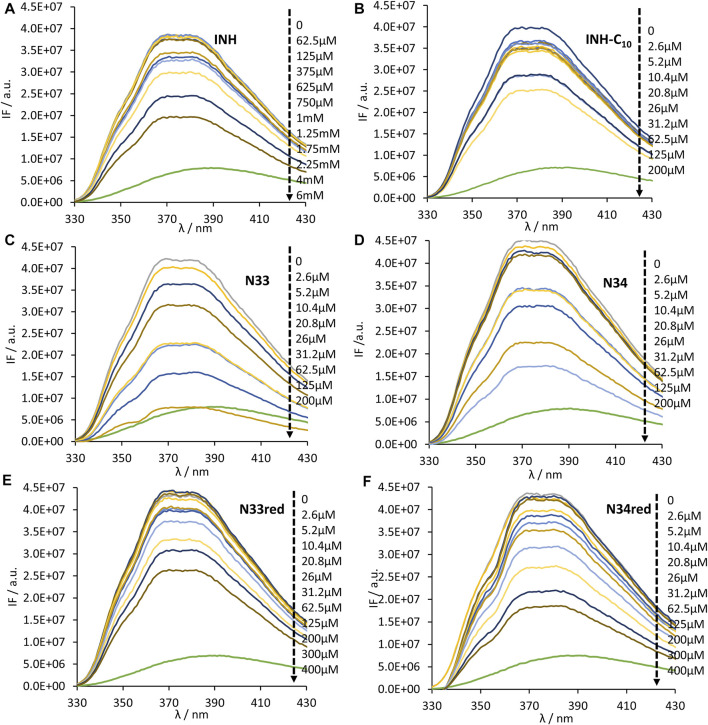
Steady-state emission spectra of the complex HSA-bound warfarin in the absence and presence of **(A)** INH, **(B)** INH-C_10_, **(C)** N33, **(D)** N34, **(E)** N33red and **(F)** N34red. λ_ex_ = 320 nm to ensure that only warfarin is excited. The green line in all plots is the emission spectrum of warfarin in water. A decrease in fluorescence intensity is observed upon compound binding (black large arrow indicates the direction of increasing compound concentration). The conditions were as follows: [warfarin] = [HSA] = 5.2 µM, kept constant; samples prepared in PBS, pH 7.4; 24 h incubation at (37.0 ± 0.5)°C; measurements at room temperature, (24.0 ± 0.5)°C.

The interaction of INH and its derivatives with HSA was further examined regarding their putative binding site. INH is known to bind to the warfarin binding pocket of site I (Wang et al., 2016). Thus, to assess if INH derivatives would bind to the same site as INH (and warfarin), fluorescence spectroscopy measurements were carried out in the presence of the fluorescent site-marker warfarin in the same molar concentration as HSA (5.2 µm). Here, to avoid any contribution from the ^214^Trp (or tyrosine residues) and ensure that only the fluorescence from warfarin was being collected, the excitation wavelength used was 320 nm (Figure 9).

As can be observed, an increase in the concentration of the compounds results in a decrease in the intensity of fluorescence emission by warfarin, which is due to the displacement of warfarin from the HSA-warfarin complex to water, where it has a much lower fluorescence quantum yield (Vasquez et al., 2009). Such decrease in the fluorescence intensity supports the hypothesis that all compounds compete with warfarin for the same binding site, or, in other words, that INH derivatives will bind at the same site as its parental compound, INH. Using the dampening in the fluorescence intensity of warfarin it is possible to retrieve the HSA-compound dissociation constant for the binding site of warfarin (*K*_dc_) - Table 3. With this purpose, the following equation, that is valid for pure competition at a single site, can be used (Cheng and Prusoff, 1973):

$$K_{\text{dc}}=\frac{C_{50}}{1+\frac{\left[\text{warfarin}\right]}{K_{\text{dw}}}}$$ (6)

where *C*_50_, the concentration of compound that displaces half of the initially bound warfarin, is determined from the variation of the fluorescence intensity, as exemplified in Supplementary Figure S9 and *K*_dw_ is the dissociation constant of warfarin. The calculated *K*_dw_ is 9 × 10^−6^ M, which is in good agreement with previous determinations (Loun and Hage, 1994; Chen and Hage, 2006; Yoo et al., 2010).

For an evaluation of the contribution of the binding to the warfarin binding site to total binding, the corresponding binding constants (the reverse of the respective dissociation constants) are presented in Supplementary Table S2. Considering these values, for the compounds that do not promote a change in the fluorescence lifetime of ^214^Trp, INH and N33red, the *K*_b_ (binding constant retrieved from the variation in the steady-state fluorescence intensity) is close to *K*_*bc*_ (binding constant for the warfarin binding site), which suggests that the main binding process of these compounds involves HSA Sudlow’s binding site I. For the other compounds, for which a variation in the fluorescence lifetime of HSA upon their binding was observed, both binding processes must be considered—the binding that induces only a decrease in the fluorescence intensity of ^214^Trp (*K*′_b_ – binding constant from the variation in the fluorescence intensity corrected regarding the variation in fluorescence lifetime) and the binding that induces a decrease in the fluorescence lifetime of ^214^Trp (*K*″_b_ – binding constant retrieved from the variation in $\bar{\tau }$ ). The detection of these two processes suggests that two binding modes are taking place, that in the case of N33 (and possibly N34) seems to comprise only warfarin binding site. In the case of N33, the outcome of the large error in the determination of *K*″_b_ is that the sum of *K*′_b_ with *K*″_b_ is not statistically different from *K*_*bc*_ and, consequently, it must be assumed that this compound is also binding solely to Sudlow’s binding site I in HSA. A similar situation occurs for N34. Despite the suggestion of two binding modes, and the identical mean values of *K*_*bc*_ and *K*′_b_, the binding to Sudlow’s site I may account for all the binding with *K*_*bc*_, due to the larger relative error in *K*′_b_ determination for this compound. For N34red the binding constant obtained from the corrected steady-state fluorescence intensity (*K*’_b_) is, within error, similar to the binding constant retrieved through competition with warfarin (*K*_*bc*_). In addition, there is a smaller contribution to binding detected from the variation of fluorescence lifetime (*K*″_b_). It is possible that these 3 compounds bind to Sudlow’s binding site I in two different orientations/different locations, in both cases displacing warfarin, but in one of them completely quenching the fluorescence emission of ^214^Trp, and in the other reducing the fluorescence lifetime. Alternatively, the binding site could accommodate one or two compound molecules, resulting in different fluorescence behaviour of ^214^Trp in each case. In the case of N34red (and to a lesser extent N34), we cannot rule-out that one of the binding processes involves a different binding site. However, this possibility becomes much more evident in the case of INH-C_10_, since the binding to Sudlow’s site I does not account for all, or even most, of the binding of the compound to HSA. In this case, there could be HSA-compound complexes 1:1 where the compound could occupy two different sites, or a stoichiometry that is not simply 1:1 but involves HSA-(compound)_n_ complexes.

**FIGURE 10:**
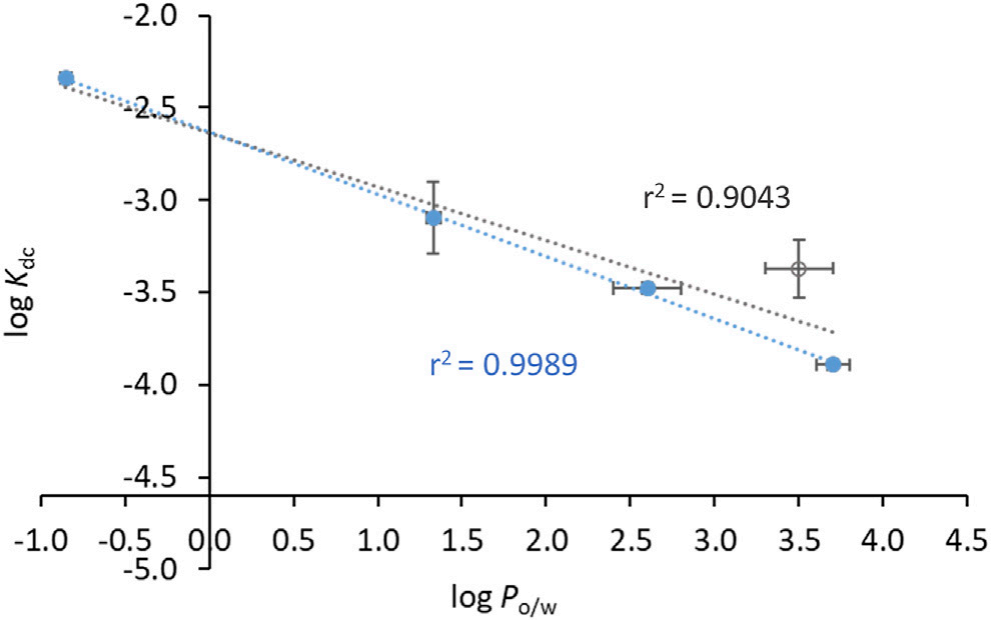
Compounds’ affinity to the binding site of warfarin in HSA (Sudlow’s binding site I) is correlated with their lipophilicity. The dots correspond to experimental data and the lines to linear fits. log P_*o/w*_ and log *K*_dc_ values can be found in Table 3. Data in closed circles are for INH, N34, N33red and N34red, and the open circle is for INH-C_10_. The linear fit either includes all the points (black line) or all points except INH-C_10_ (blue line). For the latter, the relationship between log *K*_d_ and log *P*_o/w_ is given by log *K*_dc_ = -(0.34 ± 0.01) log *P*_o/w_ - (2.63 ± 0.02).

Interestingly, the binding to the warfarin site also shows some correlation with the lipophilicity of the compounds (Figure 10). Plotting log *K*_dc_ *vs*. log *P*_o/w_ for all compounds except N33 yields a reasonably linear correlation (Figure 10, blue data). Additionally, since INH-C_10_ is the only compound for which the binding to Sudlow’s site I accounts for only a minor fraction of the binding to HSA (*K*_bc_ clearly smaller than both *K*_b_′ and *K*_b_″), we also performed a linear regression of log *K*_dc_ *vs*. log *P*_o/w_ excluding this compound (Figure 10, open circle), which significantly improved the correlation (*r* ^2^ = 0.9993).

Nonetheless, the result to be highlighted is that all derivatives studied in this work bind to warfarin binding site and all of them do it with higher affinity than INH. Since HSA is known to influence ADME processes *in vivo*, the more effective binding of the derivatives to HSA suggests that these compounds might be more efficiently transported through the blood stream, while being more protected than INH against early clearance or involvement in unintended chemical reactivity with biomolecules. In addition, the enhanced permeability and retention-like (EPR) effect, consisting in the accumulation of macromolecules such as albumin in the altered tissue, most commonly described in the context of cancer therapeutics, may be also relevant for drug targeting in tuberculosis, given the inflammatory processes associated with this infectious disease (Fenaroli et al., 2018).

### Antimycobacterial Activity

A total of six compounds were tested for their ability to inhibit the growth of actively replicating *Mtb* strains, the wild type H37Rv reference strain and a clinical strain harbouring the most common mutation on the *katG* gene, S315T. Compounds N33 and N34 are more active against the mutated strain than INH for the same strain. Compounds N33red and N34red are active against the wild-type strain, whereas their activity is completely abolished in the mutated strain. Moreover, N33 and N34 are still active against the mutated strain despite an increase in their MICs when compared with values in the wild type strain. Interestingly, for the three compounds INH-C_10_, N33 and N34, the MICs undergo a similar 20-fold increase going from *wt* to S315T, whereas for INH this increase is of approximately 150-fold. This suggests that the underlying features that render these compounds more active than INH against the mutated strain do share some similarities. All these compounds are more hydrophobic than INH (see Table 3). This can promote a better permeation of *Mtb* cell wall and plasma membrane, allowing a larger intracellular accumulation of the compounds. A previous study has shown that the hydrophobic nature of INH-C_10_ promotes a better trafficking across the *Mtb* membrane, which compensates for its smaller reactivity (the *N*′-acyl group stabilizes the molecule, resulting in slower spontaneous radical formation) when compared with INH (Vila-Viçosa et al., 2017), resulting in lower MIC values (Martins et al., 2014). The only structural difference between N33red and N34red and the parent hydrazones N33 and N34 is the nature of the bond between the *N*′ atom of the isonicotinhydrazide moiety and its substituents (Figure 1). The lack of the N′ = C bond in the reduced compounds allows the free rotation around the *N*′-C single bond in N33red and N34red. This stereo arrangement can hinder membrane permeability, *i.e*., compounds may still be adsorbed at the membrane but crossing will be much slower, thus diminishing the ability of N33red and N34red to reach and/or interact favourably with the active site of *Mtb* S315T strain, leading to higher MIC values as compared to the corresponding non-reduced compounds. The reason for the higher activity of N33 and N34 *vis-à-vis* INH in the mutant is still not totally rationalized, hinting that more than one factor is probably responsible for this behaviour (*e.g.*, a balance between trafficking and reactivity).

## Conclusion

In this work the *in vitro* toxicity and plasma protein binding of a series of isoniazid derivatives were investigated. Even though some of these INH derivatives might present a higher degree of toxicity than INH itself, they have shown to be less aggressive *in vitro* than some therapeutic agents currently in use, including the two new drugs that have been recently introduced as part of combination regimens to treat MDR-TB in adults, bedaquiline and delamanid. Although this type of comparison might have some limitations due to differences in experimental settings/conditions, the same cell type and incubation time have been employed in both cases, allowing thus a reasonable judgement about the potential safety of these derivatives. This outcome is especially relevant in the case of compounds INH-C_10_, N33 and N34, which are more active than INH against the most frequent *Mtb katG* mutation (the S315T mutant). Therefore, the rational used for the design of these INH derivatives, and their experimentally verified ability to overcome limitations caused by the S315T mutation with acceptable selectivity indexes, may be considered as strategies to improve INH activity, especially in cases where acquired resistance is emerging due to selection during treatment of a S315T mutated population.

In addition to their promising safety data, these INH derivatives also proved to have a greater binding affinity towards HSA than INH itself, with dissociation constants that in the case of INH-C_10_ are two orders of magnitude more favourable. Furthermore, the efficient binding of INH derivatives to HSA is expected to improve their half-life and solubility and lower their toxicity, allowing us to predict that in an *in vivo* scenario *Mtb* may be exposed to high and safe doses of these compounds for lengthy periods of time. The albumin binding behaviour of the studied compounds correlates with their lipophilicity, which in the case of INH-C_10_ is in harmony with prior molecular dynamic simulations predicting a higher partition to a phospholipid bilayer as opposed to INH (Vila-Viçosa et al., 2017). Nevertheless, it should be emphasized that the increased activity of N33 and N34 towards the mutated *Mtb* does not correlate with their lipophilicity. N33 and N33red have the same log P_o/w_, and although N34red is less lipophilic than N34, it still presents a higher log P_o/w_ than both N33 and N33red. However, in both cases (*i.e.*, for N33red and N34red) there is a dramatic loss of activity against the mutated *Mtb* when reducing the hydrazones to hidrazides. This is most likely related with the spatial arrangement of the molecules and their affinity towards the S1315T *katG*, as discussed above.

All summed up, we hypothesize that these INH derivatives might be able to reach and accumulate near the KatG active site more efficiently than INH, while simultaneously reducing adverse effects. These results may be of particular importance in the context of MDR-TB, for which alternatives to INH are especially urgent in order to overcome the reduced catalytic activity of the mutated KatG. The fact that some of the compounds studied here have lower MIC values against the mutant *Mtb* than INH and, simultaneously, present both a higher affinity for HSA and a potential to permeate more easily membrane barriers due to their enhanced lipophilicity, makes rather interesting to continue exploring the chemical space around INH based on the structures of the two derivatives INH-C_10_ and N34, in view of future development of new MDR-TB agents.

## Data Availability

The raw data supporting the conclusion of this article will be made available by the authors, without undue reservation.

## References

[B1] Ascenzi P. Bolli A. Di Masi A. Tundo G. R. Fanali G. Coletta M. (2011). Isoniazid and Rifampicin Inhibit Allosterically Heme Binding to Albumin and Peroxynitrite Isomerization by Heme-Albumin. J. Biol. Inorg. Chem. 16, 97–108. 10.1007/s00775-010-0706-2 20865291

[B2] Ascenzi P. Fasano M. (2010). Allostery in a Monomeric Protein: the Case of Human Serum Albumin. Biophys. Chem. 148, 16–22. 10.1016/j.bpc.2010.03.001 20346571

[B3] Bernstein J. Lott W. A. Steinberg B. A. Yale H. L. (1952). Chemotherapy of Experimental Tuberculosis. V. Isonicotinic Acid Hydrazide (Nydrazid) and Related Compounds. Am. Rev. Tuberc. 65, 357–364. 10.1164/art.1952.65.4.357 14903503

[B4] Bhadauria S. Singh G. Sinha N. Srivastava S. (2007). Isoniazid Induces Oxidative Stress, Mitochondrial Dysfunction and Apoptosis in Hep G2 Cells. Cel Mol Biol (Noisy-le-grand) 53 (1), 102–114. 10.1170/T781 17519118

[B5] Borch R. F. Bernstein M. D. Durst H. D. (1971). Cyanohydridoborate Anion as a Selective Reducing Agent. J. Am. Chem. Soc. 93, 2897–2904. 10.1021/ja00741a013

[B6] Braggio S. Montanari D. Rossi T. Ratti E. (2010). Drug Efficiency: a New Concept to Guide lead Optimization Programs towards the Selection of Better Clinical Candidates. Expert Opin. Drug Discov. 5, 609–618. 10.1517/17460441.2010.490553 22823203

[B7] Brigden G. Hewison C. Varaine F. (2015). New Developments in the Treatment of Drug-Resistant Tuberculosis: Clinical Utility of Bedaquiline and Delamanid. Infect. Drug Resist. 8, 367–378. 10.2147/IDR.S68351 26586956 PMC4634826

[B8] Canetti G. Froman S. Grosset J. Hauduroy P. Langerova M. Mahler H. T. (1963). Mycobacteria: Laboratory Methods for Testing Drug Sensitivity and Resistance. Bull. World Health Organ. 29, 565–578. 14102034 PMC2555065

[B9] Chen J. Hage D. S. (2006). Quantitative Studies of Allosteric Effects by Biointeraction Chromatography: Analysis of Protein Binding for Low-Solubility Drugs. Anal. Chem. 78, 2672–2683. 10.1021/ac052017b 16615779 PMC2556871

[B10] Cheng Y. Prusoff W. H. (1973). Relationship between the Inhibition Constant (K1) and the Concentration of Inhibitor Which Causes 50 Per Cent Inhibition (I50) of an Enzymatic Reaction. Biochem. Pharmacol. 22, 3099–3108. 10.1016/0006-2952(73)90196-2 4202581

[B11] Colmenarejo G. (2003). In Silico prediction of Drug-Binding Strengths to Human Serum Albumin. Med. Res. Rev. 23, 275–301. 10.1002/med.10039 12647311

[B12] Croom E. (2012). “Chapter Three - Metabolism of Xenobiotics of Human Environments,” in Progress in Molecular Biology and Translational Science. Editor HODGSON E. (Cambridge, MA, United States: Academic Press). 10.1016/B978-0-12-415813-9.00003-9 22974737

[B13] Curry S. Mandelkow H. Brick P. Franks N. (1998). Crystal Structure of Human Serum Albumin Complexed with Fatty Acid Reveals an Asymmetric Distribution of Binding Sites. Nat. Struct. Biol. 5, 827–835. 10.1038/1869 9731778

[B14] Demoro B. De Almeida R. F. Marques F. Matos C. P. Otero L. Costa Pessoa J. (2013). Screening Organometallic Binuclear Thiosemicarbazone Ruthenium Complexes as Potential Anti-tumour Agents: Cytotoxic Activity and Human Serum Albumin Binding Mechanism. Dalton Trans. 42, 7131–7146. 10.1039/c3dt00028a 23519281

[B15] Devarbhavi H. (2012). An Update on Drug-Induced Liver Injury. J. Clin. Exp. Hepatol. 2, 247–259. 10.1016/j.jceh.2012.05.002 25755441 PMC3940315

[B16] Dockal M. Carter D. C. Rüker F. (1999). The Three Recombinant Domains of Human Serum Albumin. Structural Characterization and Ligand Binding Properties. J. Biol. Chem. 274, 29303–29310. 10.1074/jbc.274.41.29303 10506189

[B17] Fenaroli F. Repnik U. Xu Y. Johann K. Van Herck S. Dey P. (2018). Enhanced Permeability and Retention-like Extravasation of Nanoparticles from the Vasculature into Tuberculosis Granulomas in Zebrafish and Mouse Models. ACS Nano 12, 8646–8661. 10.1021/acsnano.8b04433 30081622

[B18] Frötschl R. Weickardt S. Staszewski S. Kaufmann G. Kasper P. (2005). Effects of Chlorpromazine with and without UV Irradiation on Gene Expression of HepG2 Cells. Mutat. Res. 575, 47–60. 10.1016/j.mrfmmm.2005.03.002 15924885

[B19] Gandhi A. Guo T. Shah P. Moorthy B. Ghose R. (2013). Chlorpromazine-induced Hepatotoxicity during Inflammation Is Mediated by TIRAP-dependent Signaling Pathway in Mice. Toxicol. Appl. Pharmacol. 266, 430–438. 10.1016/j.taap.2012.11.030 23238562 PMC3849342

[B20] Girling D. J. (1978). The Hepatic Toxicity of Antituberculosis Regimens Containing Isoniazid, Rifampicin and Pyrazinamide. Tubercle 59, 13–32. 10.1016/0041-3879(77)90022-8 345572

[B21] Hazbón M. H. Brimacombe M. Bobadilla Del Valle M. Cavatore M. Guerrero M. I. Varma-Basil M. (2006). Population Genetics Study of Isoniazid Resistance Mutations and Evolution of Multidrug-Resistant *Mycobacterium tuberculosis* . Antimicrob. Agents Chemother. 50, 2640–2649. 10.1128/AAC.00112-06 16870753 PMC1538650

[B22] Heym B. Zhang Y. Poulet S. Young D. Cole S. T. (1993). Characterization of the katG Gene Encoding a Catalase-Peroxidase Required for the Isoniazid Susceptibility of *Mycobacterium tuberculosis* . J. Bacteriol. 175, 4255–4259. 10.1128/jb.175.13.4255-4259.1993 8320241 PMC204858

[B23] Huang Y. S. Chern H. D. Su W. J. Wu J. C. Chang S. C. Chiang C. H. (2003). Cytochrome P450 2E1 Genotype and the Susceptibility to Antituberculosis Drug-Induced Hepatitis. Hepatology 37, 924–930. 10.1053/jhep.2003.50144 12668988

[B24] Jakštys B. Ruzgys P. Tamošiūnas M. Šatkauskas S. (2015). Different Cell Viability Assays Reveal Inconsistent Results after Bleomycin Electrotransfer *In Vitro* . J. Membr. Biol. 248, 857–863. 10.1007/s00232-015-9813-x 26077843

[B25] Joseph K. S. Anguizola J. Hage D. S. (2011). Binding of Tolbutamide to Glycated Human Serum Albumin. J. Pharm. Biomed. Anal. 54, 426–432. 10.1016/j.jpba.2010.09.003 20880646 PMC2962718

[B26] Joseph K. S. Anguizola J. Jackson A. J. Hage D. S. (2010). Chromatographic Analysis of Acetohexamide Binding to Glycated Human Serum Albumin. J. Chromatogr. B Analyt Technol. Biomed. Life Sci. 878, 2775–2781. 10.1016/j.jchromb.2010.08.021 PMC2952882 20829128

[B27] Kanakis C. D. Tarantilis P. A. Polissiou M. G. Diamantoglou S. Tajmir-Riahi H. A. (2006). Antioxidant Flavonoids Bind Human Serum Albumin. J. Mol. Struct. 798, 69–74. 10.1016/j.molstruc.2006.03.051

[B28] Keam S. J. (2019). Pretomanid: First Approval. Drugs 79, 1797–1803. 10.1007/s40265-019-01207-9 31583606

[B29] Kim H. S. Wainer I. W. (2008). Rapid Analysis of the Interactions between Drugs and Human Serum Albumin (HSA) Using High-Performance Affinity Chromatography (HPAC). J. Chromatogr. B Analyt Technol. Biomed. Life Sci. 870, 22–26. 10.1016/j.jchromb.2008.05.029 PMC2556154 18554995

[B30] Kratz F. Elsadek B. (2012). Clinical Impact of Serum Proteins on Drug Delivery. J. Control. Release 161, 429–445. 10.1016/j.jconrel.2011.11.028 22155554

[B31] Lakowicz J. R. (2006). Principles of Fluorescence Spectroscopy. New York, USA: Springer Science.

[B32] Loun B. Hage D. S. (1994). Chiral Separation Mechanisms in Protein-Based HPLC Columns. 1. Thermodynamic Studies of (R)- and (S)-warfarin Binding to Immobilized Human Serum Albumin. Anal. Chem. 66, 3814–3822. 10.1021/ac00093a043 7802261

[B33] Luís D. V. Silva J. Tomaz A. I. De Almeida R. F. Larguinho M. Baptista P. V. (2014). Insights into the Mechanisms Underlying the Antiproliferative Potential of a Co(II) Coordination Compound Bearing 1,10-Phenanthroline-5,6-Dione: DNA and Protein Interaction Studies. J. Biol. Inorg. Chem. 19, 787–803. 10.1007/s00775-014-1110-0 24481501

[B34] Lupien A. Vocat A. Foo C. S. Blattes E. Gillon J. Y. Makarov V. (2018). Optimized Background Regimen for Treatment of Active Tuberculosis with the Next-Generation Benzothiazinone Macozinone (PBTZ169). Antimicrob. Agents Chemother. 62 (11), e00840–18. 10.1128/AAC.00840-18 30126954 PMC6201121

[B35] Machado D. Girardini M. Viveiros M. Pieroni M. (2018). Challenging the Drug-Likeness Dogma for New Drug Discovery in Tuberculosis. Front Microbiol. 9, 1367. 10.3389/fmicb.2018.01367 30018597 PMC6037898

[B36] Machado D. Pires D. Perdigão J. Couto I. Portugal I. Martins M. (2016). Ion Channel Blockers as Antimicrobial Agents, Efflux Inhibitors, and Enhancers of Macrophage Killing Activity against Drug Resistant *Mycobacterium tuberculosis* . PloS one 11 (2), e0149326. 10.1371/journal.pone.0149326 26919135 PMC4769142

[B37] Maiti T. K. Ghosh K. S. Debnath J. Dasgupta S. (2006). Binding of All-Trans Retinoic Acid to Human Serum Albumin: Fluorescence, FT-IR and Circular Dichroism Studies. Int. J. Biol. Macromol 38, 197–202. 10.1016/j.ijbiomac.2006.02.015 16569428

[B38] Mallik R. Yoo M. J. Chen S. Hage D. S. (2008). Studies of Verapamil Binding to Human Serum Albumin by High-Performance Affinity Chromatography. J. Chromatogr. B Analyt Technol. Biomed. Life Sci. 876, 69–75. 10.1016/j.jchromb.2008.10.022 PMC2597894 18980867

[B39] Martins F. Santos S. Ventura C. Elvas-Leitão R. Santos L. Vitorino S. (2014). Design, Synthesis and Biological Evaluation of Novel Isoniazid Derivatives with Potent Antitubercular Activity. Eur. J. Med. Chem. 81, 119–138. 10.1016/j.ejmech.2014.04.077 24836065

[B40] Matos C. P. Valente A. Marques F. Adão P. Paula Robalo M. De Almeida R. F. M. (2013). New Polydentate Ru(III)-Salan Complexes: Synthesis, Characterization, Anti-tumour Activity and Interaction with Human Serum Proteins. Inorg. Chim. Acta 394, 616–626. 10.1016/j.ica.2012.09.026

[B41] Matsuda R. Anguizola J. Joseph K. S. Hage D. S. (2011). High-performance Affinity Chromatography and the Analysis of Drug Interactions with Modified Proteins: Binding of Gliclazide with Glycated Human Serum Albumin. Anal. Bioanal. Chem. 401, 2811–2819. 10.1007/s00216-011-5382-8 21922305 PMC3205319

[B42] Mishra P. Singh U. Pandey C. M. Mishra P. Pandey G. (2019). Application of Student's T-Test, Analysis of Variance, and Covariance. Ann. Card. Anaesth. 22 (4), 407–411. 10.4103/aca.ACA_94_19 31621677 PMC6813708

[B43] Morgan K. Martucci N. Kozlowska A. Gamal W. Brzeszczyński F. Treskes P. (2019). Chlorpromazine Toxicity Is Associated with Disruption of Cell Membrane Integrity and Initiation of a Pro-inflammatory Response in the HepaRG Hepatic Cell Line. Biomed. Pharmacother. 111, 1408–1416. 10.1016/j.biopha.2019.01.020 30841456

[B44] Muller P. Y. Milton M. N. (2012). The Determination and Interpretation of the Therapeutic index in Drug Development. Nat. Rev. Drug Discov. 11, 751–761. 10.1038/nrd3801 22935759

[B45] Niepel M. Hafner M. Mills C. E. Subramanian K. Williams E. H. Chung M. (2019). A Multi-center Study on the Reproducibility of Drug-Response Assays in Mammalian Cell Lines. Cell Syst 9, 35–48. e5. 10.1016/j.cels.2019.06.005 31302153 PMC6700527

[B46] Ong C. W. M. Migliori G. B. Raviglione M. Macgregor-Skinner G. Sotgiu G. Alffenaar J. W. (2020). Epidemic and Pandemic Viral Infections: Impact on Tuberculosis and the Lung: A Consensus by the World Association for Infectious Diseases and Immunological Disorders (WAidid), Global Tuberculosis Network (GTN), and Members of the European Society of Clinical Microbiology and Infectious Diseases Study Group for Mycobacterial Infections (ESGMYC). Eur. Respir. J. 56, 2001727. 10.1183/13993003.01727-2020 32586885 PMC7527651

[B47] Pace C. N. Vajdos F. Fee L. Grimsley G. Gray T. (1995). How to Measure and Predict the Molar Absorption Coefficient of a Protein. Protein Sci. 4, 2411–2423. 10.1002/pro.5560041120 8563639 PMC2143013

[B48] Pellegatti M. Pagliarusco S. Solazzo L. Colato D. (2011). Plasma Protein Binding and Blood-free Concentrations: Which Studies Are Needed to Develop a Drug? Expert Opin. Drug Metab. Toxicol. 7, 1009–1020. 10.1517/17425255.2011.586336 21635153

[B49] Pessoa J. C. Tomaz I. (2010). Transport of Therapeutic Vanadium and Ruthenium Complexes by Blood Plasma Components. Curr. Med. Chem. 17, 3701–3738. 10.2174/092986710793213742 20846109

[B50] Petitpas I. Bhattacharya A. A. Twine S. East M. Curry S. (2001). Crystal Structure Analysis of Warfarin Binding to Human Serum Albumin: Anatomy of Drug Site I. J. Biol. Chem. 276, 22804–22809. 10.1074/jbc.M100575200 11285262

[B51] Pinheiro M. Silva A. S. Pisco S. Reis S. (2014). Interactions of Isoniazid with Membrane Models: Implications for Drug Mechanism of Action. Chem. Phys. Lipids 183, 184–190. 10.1016/j.chemphyslip.2014.07.002 25016155

[B52] Ràfols C. Bosch E. Ruiz R. Box K. J. Reis M. Ventura C. (2012). Acidity and Hydrophobicity of Several New Potential Antitubercular Drugs: Isoniazid and Benzimidazole Derivatives. J. Chem. Eng. Data 57, 330–338.

[B53] Rawat R. Whitty A. Tonge P. J. (2003). The Isoniazid-NAD Adduct Is a Slow, Tight-Binding Inhibitor of InhA, the *Mycobacterium tuberculosis* Enoyl Reductase: Adduct Affinity and Drug Resistance. Proc. Natl. Acad. Sci. U S A. 100, 13881–13886. 10.1073/pnas.2235848100 14623976 PMC283515

[B54] Riss T. L. M. Niles A. L. Duellman S. Benink H. A. Worzella T. J. Minor L. (2013). “Cell Viability Assays,” in Assay Guidance Manual. Markossian S. S. Grossman A. Brimacombe K.. Editors (Bethesda (MD): Eli Lilly & Company and the National Center for Advancing Translational Sciences).

[B55] Santos M. S. C. S. Matos A. M. Reis M. Martins F. (2020). Lipophilicity Assessment of Some Isoniazid Derivatives Active against *Mycobacterium tuberculosis* . Colloids Surf. A: Physicochemical Eng. Aspects 599, 124820. 10.1016/j.colsurfa.2020.124820

[B56] Schmidt S. Gonzalez D. Derendorf H. (2010). Significance of Protein Binding in Pharmacokinetics and Pharmacodynamics. J. Pharm. Sci. 99, 1107–1122. 10.1002/jps.21916 19852037

[B57] Singh M. Sasi P. Rai G. Gupta V. H. Amarapurkar D. Wangikar P. P. (2011). Studies on Toxicity of Antitubercular Drugs Namely Isoniazid, Rifampicin, and Pyrazinamide in an *In Vitro* Model of HepG2 Cell Line. Med. Chem. Res. 20, 1611–1615. 10.1007/s00044-010-9405-3

[B58] Smith D. A. Di L. Kerns E. H. (2010). The Effect of Plasma Protein Binding on *In Vivo* Efficacy: Misconceptions in Drug Discovery. Nat. Rev. Drug Discov. 9, 929–939. 10.1038/nrd3287 21119731

[B59] Sowell J. Mason J. C. Strekowski L. Patonay G. (2001). Binding Constant Determination of Drugs toward Subdomain IIIA of Human Serum Albumin by Near-Infrared Dye-Displacement Capillary Electrophoresis. Electrophoresis 22, 2512–2517. 10.1002/1522-2683(200107)22:12<2512::AID-ELPS2512>3.0.CO;2-9 11519955

[B60] Springer B. Lucke K. Calligaris-Maibach R. Ritter C. Böttger E. C. (2009). Quantitative Drug Susceptibility Testing of *Mycobacterium tuberculosis* by Use of MGIT 960 and EpiCenter Instrumentation. J. Clin. Microbiol. 47 (6), 1773–1780. 10.1128/JCM.02501-08 19339475 PMC2691107

[B61] Starosta R. Santos F. C. De Almeida R. F. M. (2020). Human and Bovine Serum Albumin Time-Resolved Fluorescence: Tryptophan and Tyrosine Contributions, Effect of DMSO and Rotational Diffusion. J. Mol. Struct. 1221, 128805. 10.1016/j.molstruc.2020.128805

[B62] Sudlow G. Birkett D. J. Wade D. N. (1976). Further Characterization of Specific Drug Binding Sites on Human Serum Albumin. Mol. Pharmacol. 12, 1052–1061. 1004490

[B63] Teale F. W. Weber G. (1957). Ultraviolet Fluorescence of the Aromatic Amino Acids. Biochem. J. 65, 476–482. 10.1042/bj0650476 13412650 PMC1199900

[B64] Teixeira V. H. Ventura C. Leitão R. Ràfols C. Bosch E. Martins F. (2015). Molecular Details of INH-C10 Binding to Wt KatG and its S315T Mutant. Mol. Pharm. 12, 898–909. 10.1021/mp500736n 25590860

[B65] Vahdati-Mashhadian N. Jafari M. R. Sharghi N. Sanati T. (2013). Protective Effects of Vitamin C and NAC on the Toxicity of Rifampin on Hepg2 Cells. Iran J. Pharm. Res. 12, 141–146. 24250582 PMC3813199

[B66] Vasquez J. M. Vu A. Schultz J. S. Vullev V. I. (2009). Fluorescence Enhancement of Warfarin Induced by Interaction with Beta-Cyclodextrin. Biotechnol. Prog. 25, 906–914. 10.1002/btpr.188 19455641

[B67] Vila-Viçosa D. Victor B. L. Ramos J. Machado D. Viveiros M. Switala J. (2017). Insights on the Mechanism of Action of INH-C10 as an Antitubercular Prodrug. Mol. Pharm. 14, 4597–4605. 10.1021/acs.molpharmaceut.7b00719 29091448

[B68] Vilchèze C. Jacobs W. R. (2007). The Mechanism of Isoniazid Killing: Clarity through the Scope of Genetics. Annu. Rev. Microbiol. 61, 35–50. 10.1146/annurev.micro.61.111606.122346 18035606

[B69] Wanat K. (2020). Biological Barriers, and the Influence of Protein Binding on the Passage of Drugs across Them. Mol. Biol. Rep. 47, 3221–3231. 10.1007/s11033-020-05361-2 32140957

[B70] Wang K. Shindoh H. Inoue T. Horii I. (2002). Advantages of *In Vitro* Cytotoxicity Testing by Using Primary Rat Hepatocytes in Comparison with Established Cell Lines. J. Toxicol. Sci. 27, 229–237. 10.2131/jts.27.229 12238146

[B71] Wang Y. R. Fang Q. Hu T. Y. Liu Y. (2016). Spectroscopic and Molecular Docking Study on Specific Binding and Inhibition of Isoniazid to Human Serum Albumin and Catalase. Guang Pu Xue Yu Guang Pu Fen Xi 36, 3789–3795. 30226718

[B72] Winder F. G. Collins P. B. (1970). Inhibition by Isoniazid of Synthesis of Mycolic Acids in *Mycobacterium tuberculosis* . J. Gen. Microbiol. 63, 41–48. 10.1099/00221287-63-1-41 5500025

[B73] Wiseman B. Carpena X. Feliz M. Donald L. J. Pons M. Fita I. (2010). Isonicotinic Acid Hydrazide Conversion to Isonicotinyl-NAD by Catalase-Peroxidases. J. Biol. Chem. 285, 26662–26673. 10.1074/jbc.M110.139428 20554537 PMC2924108

[B74] World Health Organization (2021). Global Tuberculosis Report 2021. Available at: https://www.who.int/publications/i/item/9789240037021 .

[B75] Wu D. Cederbaum A. I. (1996). Ethanol Cytotoxicity to a Transfected HepG2 Cell Line Expressing Human Cytochrome P4502E1. J. Biol. Chem. 271, 23914–23919. 10.1074/jbc.271.39.23914 8798623

[B76] Yang F. Zhang Y. Liang H. (2014). Interactive Association of Drugs Binding to Human Serum Albumin. Int. J. Mol. Sci. 15, 3580–3595. 10.3390/ijms15033580 24583848 PMC3975355

[B77] Yoo M. J. Schiel J. E. Hage D. S. (2010). Evaluation of Affinity Microcolumns Containing Human Serum Albumin for Rapid Analysis of Drug-Protein Binding. J. Chromatogr. B Analyt Technol. Biomed. Life Sci. 878, 1707–1713. 10.1016/j.jchromb.2010.04.028 PMC2878846 20462808

[B78] Zhao X. Yu H. Yu S. Wang F. Sacchettini J. C. Magliozzo R. S. (2006). Hydrogen Peroxide-Mediated Isoniazid Activation Catalyzed by *Mycobacterium tuberculosis* Catalase-Peroxidase (KatG) and its S315T Mutant. Biochemistry 45, 4131–4140. 10.1021/bi051967o 16566587

[B79] Zumla A. Chakaya J. Centis R. D'Ambrosio L. Mwaba P. Bates M. (2015). Tuberculosis Treatment and Management-Aan Update on Treatment Regimens, Trials, New Drugs, and Adjunct Therapies. Lancet Respir. Med. 3, 220–234. 10.1016/S2213-2600(15)00063-6 25773212

