# Supplementary material for: In vitro Evaluation of Isoniazid Derivatives as Potential Agents Against Drug-Resistant Tuberculosis
Source: Front Pharmacol. 2022 May 4;13:868545. doi: 10.3389/fphar.2022.868545 (PMC9114799; doi:10.3389/fphar.2022.868545)
Supplement: Supplementary file 1 [file DataSheet1.pdf]

Supplementary Material

In vitro evaluation of isoniazid derivatives as potential agents against drug-resistant tuberculosis

Joaquim Trigo Marquês[1], Catarina Frazão de Faria[1], Marina Reis[1,2], Diana Machado[3], Susana Santos[1], Maria da Soledade Santos[1], Miguel Viveiros[3], Filomena Martins[1*], Rodrigo F. M. de Almeida[1*]

[1]Centro de Química Estrutural, Institute of Molecular Sciences, Departamento de Química e Bioquímica, Faculdade de Ciências, Universidade de Lisboa, 1749-016 Lisboa, Portugal

[2] Instituto Superior de Educação e Ciências (ISEC Lisboa), Alameda das Linhas de Torres, 174, 1750-142 Lisboa, Portugal

[3] Unidade de Microbiologia Medica, Global Health and Tropical Medicine, Instituto de Higiene e Medicina Tropical, Universidade Nova de Lisboa, Rua da Junqueira 100, 1349-008 Lisboa, Portugal

*Corresponding authors: filomena.martins @fc.ul.pt; rfalmeida@fc.ul.pt

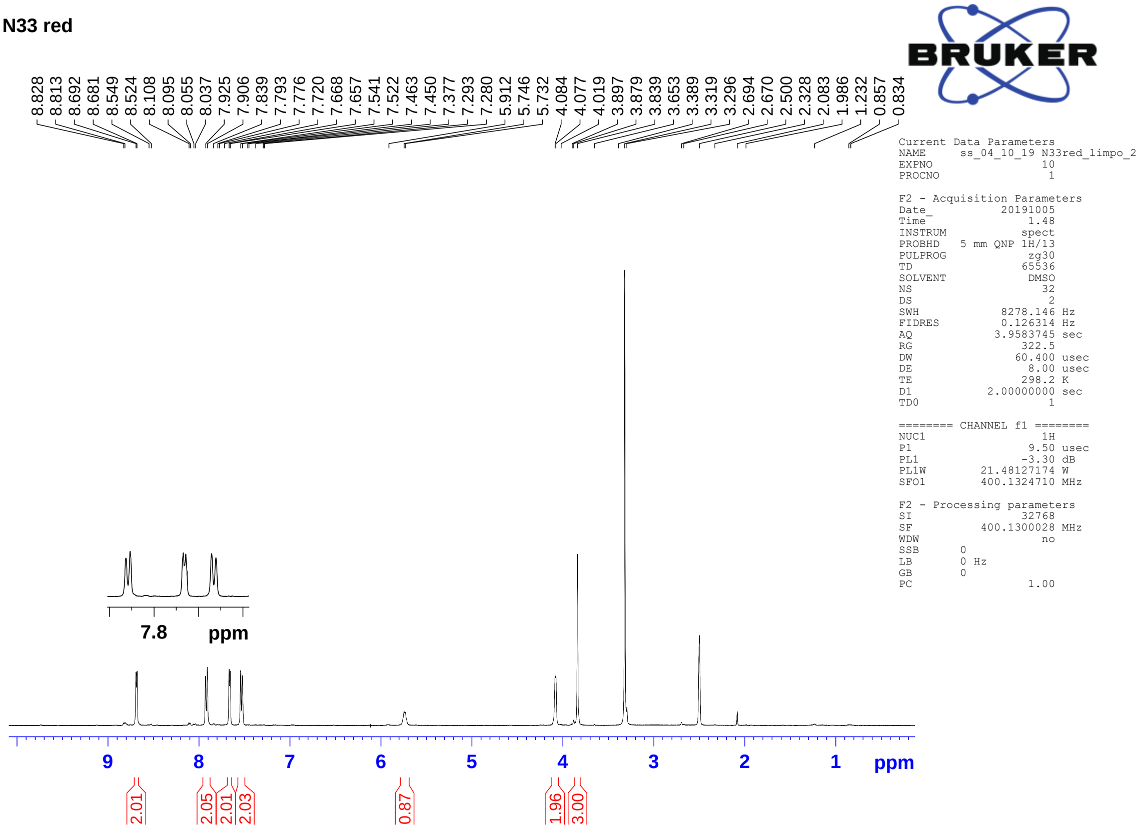

Figure S1 – ^{1}H NMR of N33red

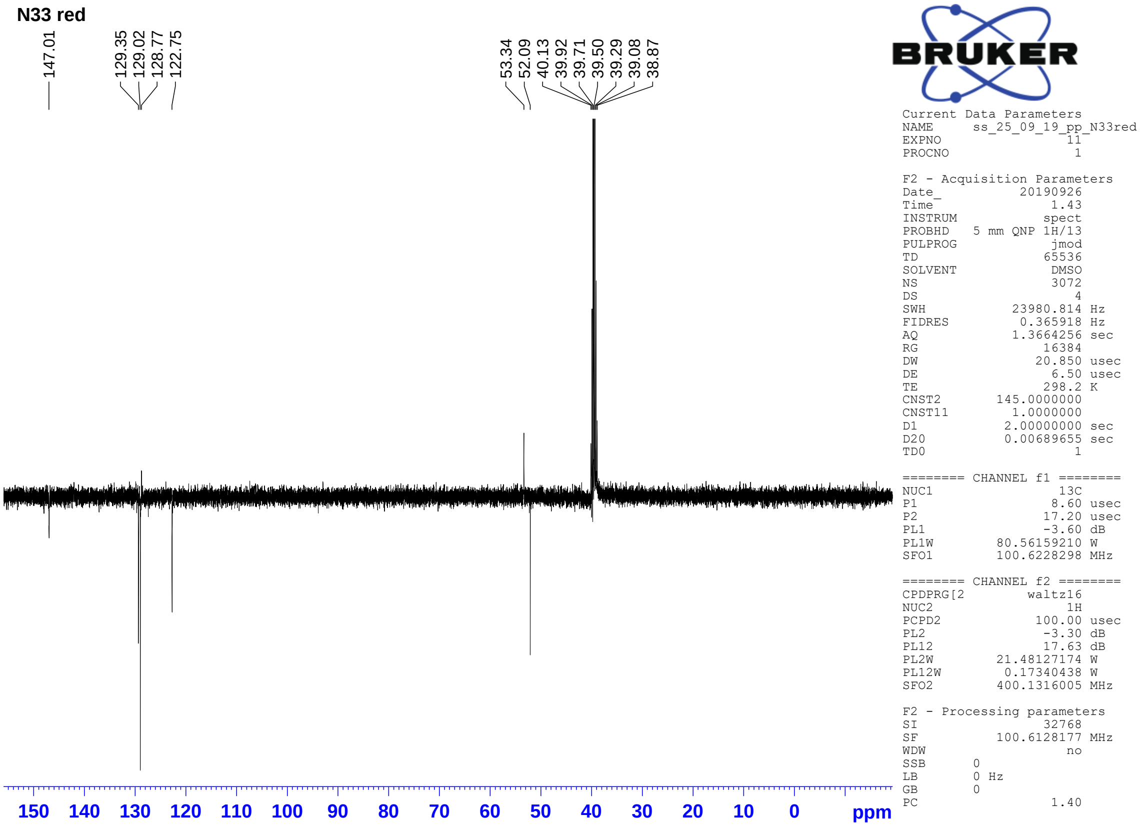

Figure S2 – ^{13}C NMR APT of N33red

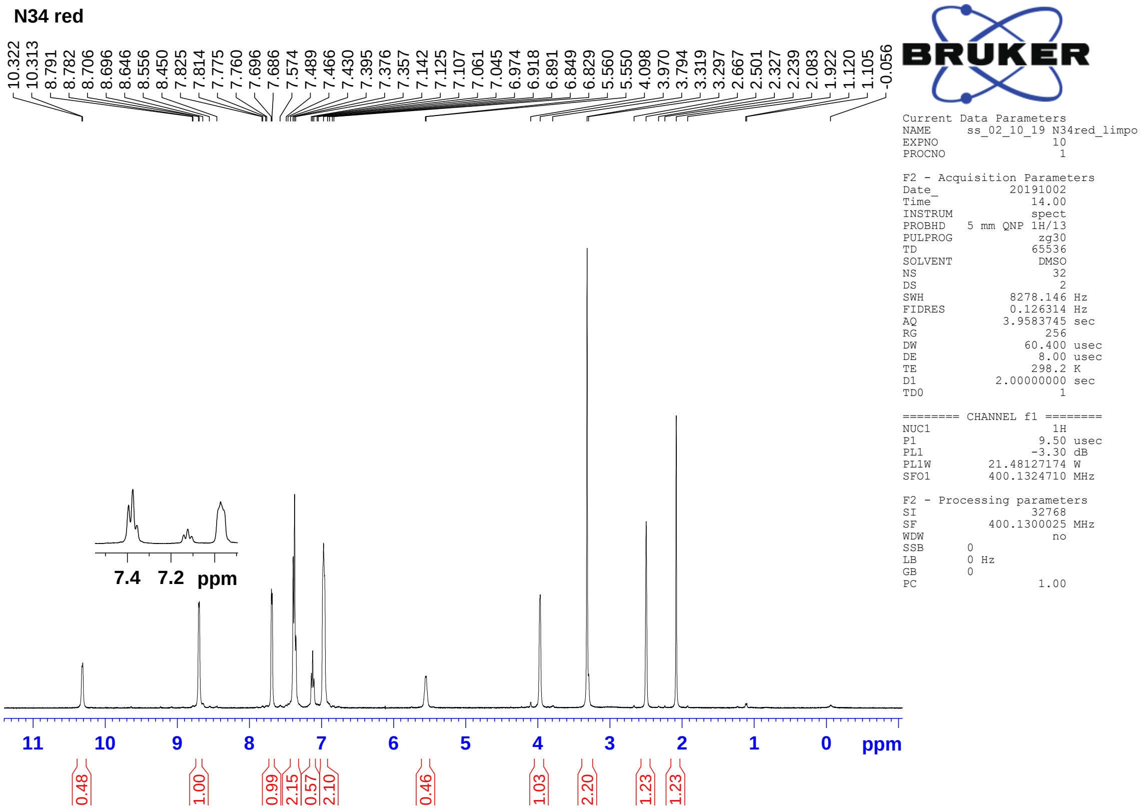

Figure S3 – ^{1}H NMR of N34red

N34 red

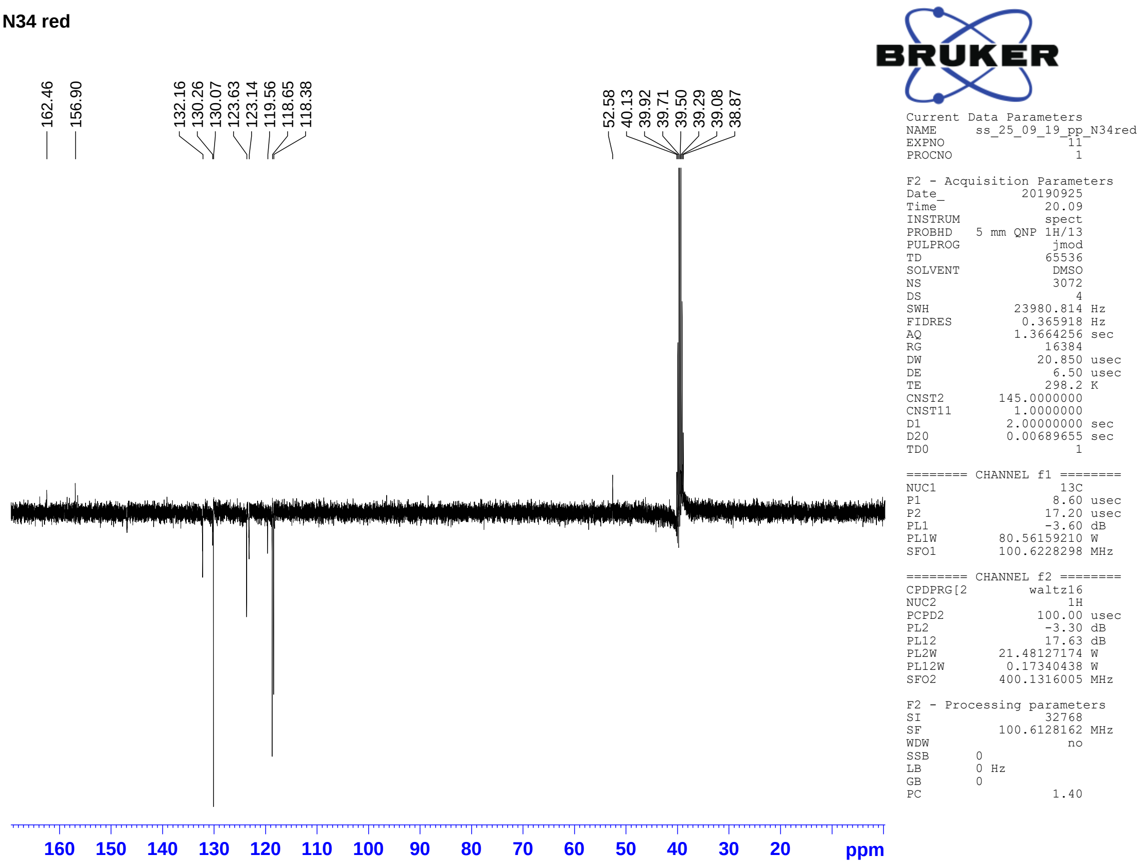

Figure S4 – ^{13}C NMR APT of N34red

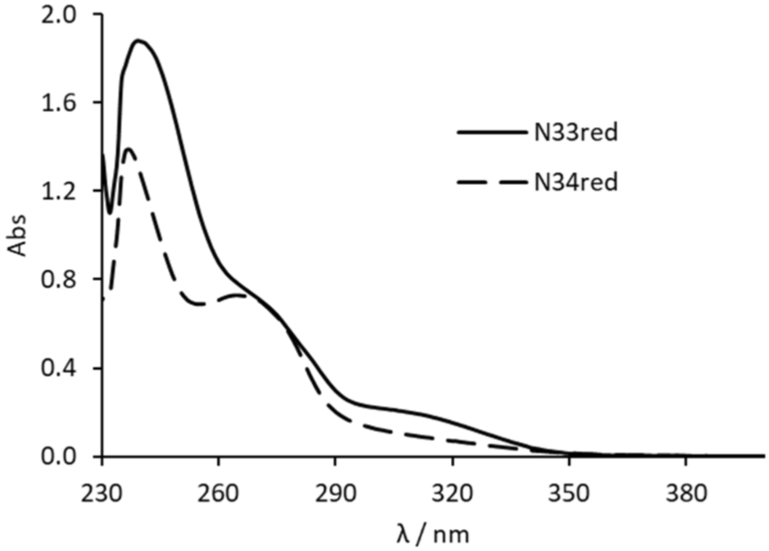

Figure S5 – Electronic absorption spectra of N33red and N34red in PBS buffer pH 7.4 with 5 % DMSO were monitored over 3 days. The fluctuation of the spectra in the UV region of N33red at 240 nm was less than 1.2% while for N34red at 269 nm was less than 2.5%. The time dependence of the absorbance readings did not present a systematic pattern and the observed fluctuations are below the measurement uncertainty, thus confirming the stability of both compounds for at least 3 days.

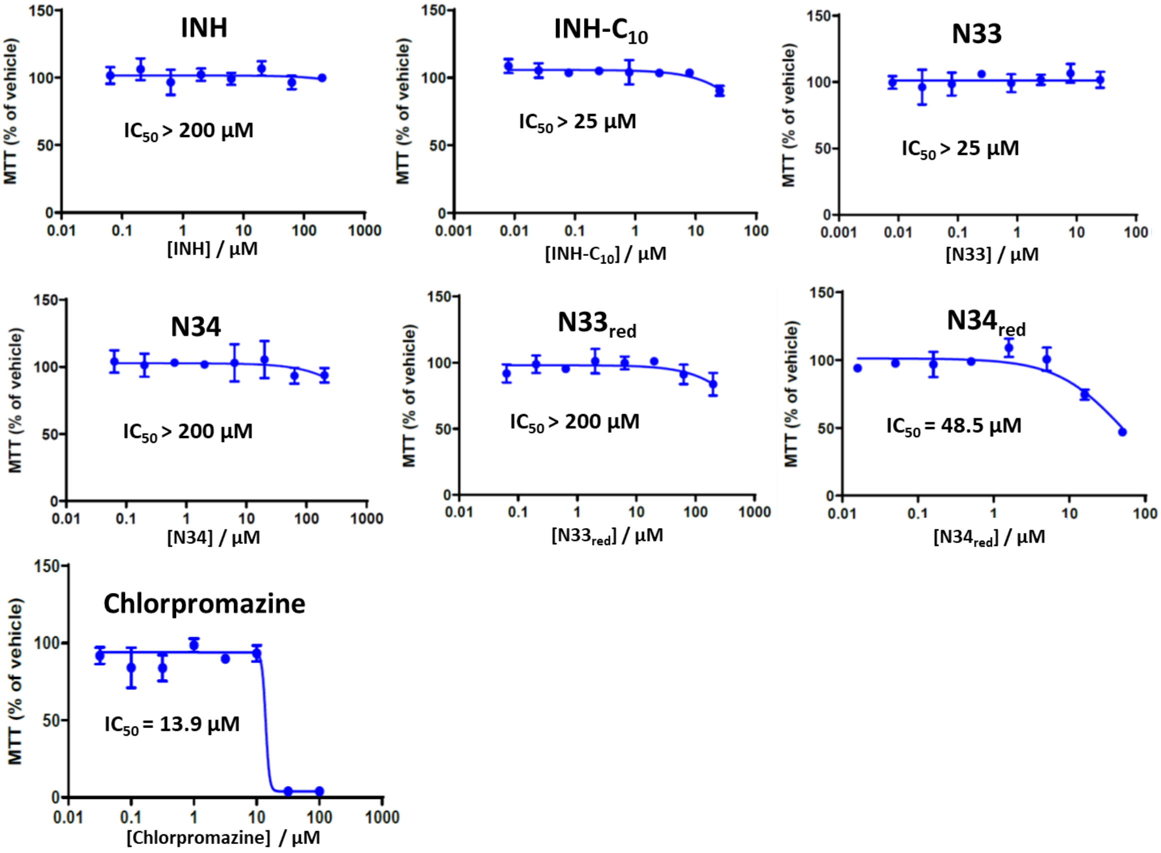

Figure S6 – Cell viability assays for INH and its derivatives and the positive control, chlorpromazine. Solubility issues limited the top concentration assessed in each case. HepG2 cells were incubated with the compounds for 72 h at 37 °C. Vehicle: DMSO at a final concentration of 0.5%. Further experimental details in the main text.

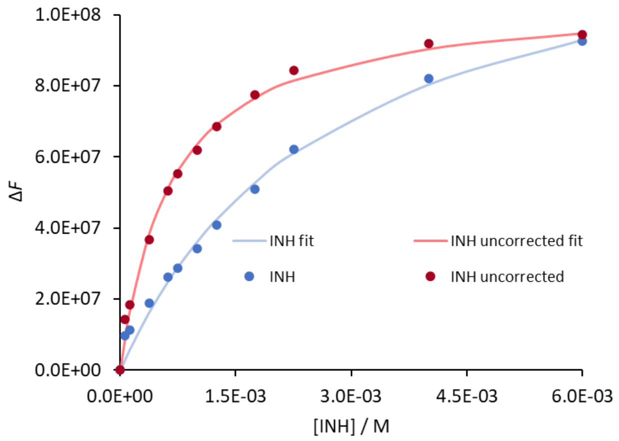

Figure S7 – INH binding to HSA assessed by steady-state fluorescence spectroscopy: fluorescence intensity values (λ_{ex} = 295 nm, λ_{em} = 340 nm) corrected for inner filter effects (blue dots) and uncorrected (red dots) and respective nonlinear fit of equation 5 (blue and red lines, respectively). The dissociation constants retrieved for these illustrative sets of data are 2.72 × 10^{-3} M (corrected fluorescence) and 6.5 × 10^{-4} M (uncorrected fluorescence). Other experimental conditions: [HSA] = 5.2 µM; PBS, pH 7.4; 24 h incubation at (37.0 ± 0.5) °C; measurements at (24.0 ± 0.5) °C.

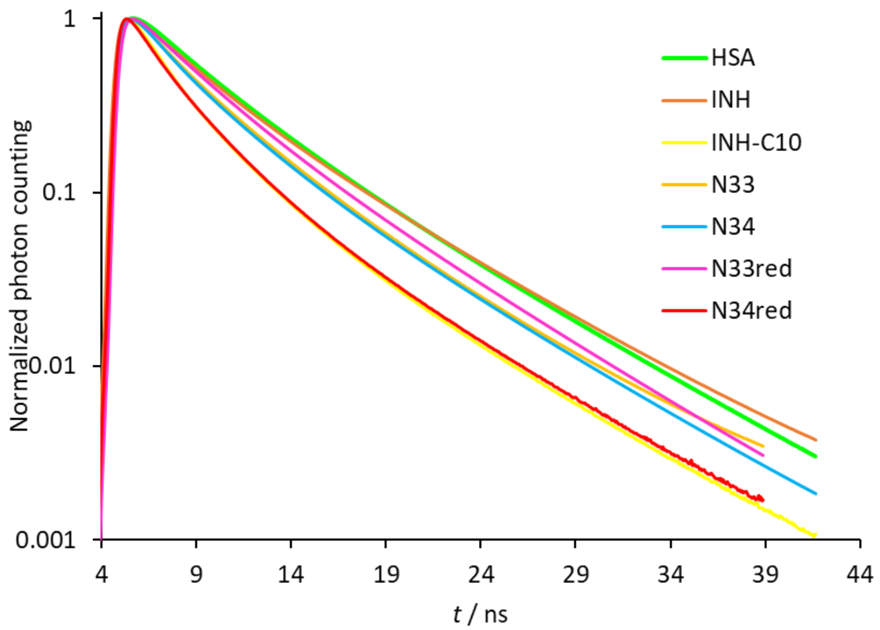

Figure S8 – Normalized fluorescence intensity decays of HSA ^{214}Trp in the absence and presence of a) INH, b) INH-C10, c) N33, d) N34, e) N33red and f) N34red, with excitation at 279 nm and emission at 350 nm (thus avoiding the emission of tyrosine residues). Other experimental conditions: [HSA] = 5.2 µM, kept constant; samples prepared in PBS, pH 7.4; 24 h incubation at (37.0 ± 0.5) °C; measurements at (24.0 ± 0.5) °C; compound concentrations: the highest used in this study (Table 1 in the main text).

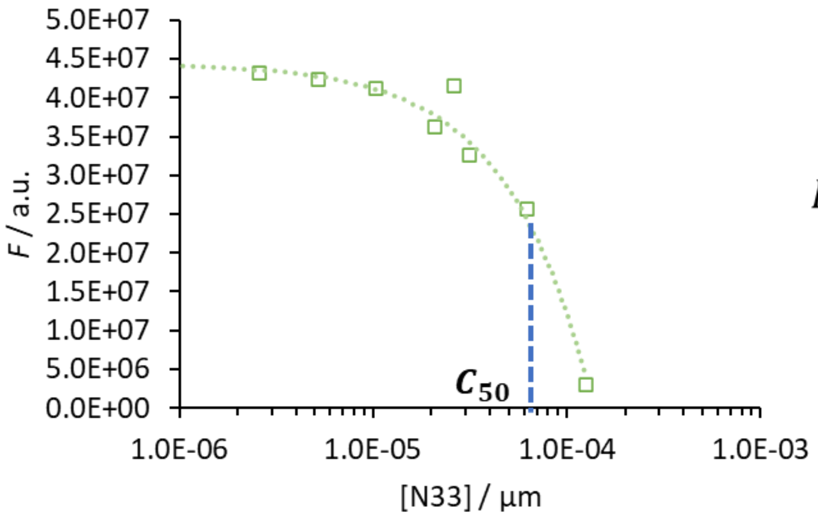

$$K_{dc} = \frac{C_{50}}{1 + \frac{[\text{warfarin}]}{K_{dw}}}$$

Figure S9 – Variation in the steady-state fluorescence intensity of warfarin with increasing amounts of N33. The decrease in fluorescence intensity of warfarin (λ_{exc} = 320 nm) is observed as the concentration of N33 increases and warfarin is displaced from HSA Sudlow's binding site I into water. From this variation it is possible to retrieve C_{50}, the concentration of compound that displaces half of the initially bound warfarin. This concentration is given by the fluorescence intensity corresponding to (F^{bound} – F^{free})/2, where F^{bound} is the intensity of warfarin in the absence of the compound and F^{free} is the intensity of warfarin in water (buffer), which was obtained for warfarin in PBS in the absence of HSA. The C_{50} value was retrieved for each replicate and the average was used to calculate the K_{dc} of each compound. An illustrative example of how C_{50} was retrieved is shown in the plot for one of the replicates of N33. Other experimental conditions: [HSA] = [warfarin] = 5.2 µM, kept constant; samples prepared in PBS, pH 7.4; 24 h incubation at (37.0 ± 0.5) °C; measurements at room temperature, (24.0 ± 0.5) °C.

Table S1 – IC_{50} values of the compounds studied in this work expressed in μg mL^{-1} against HepG2 cells, and MIC values against *wt* (MIC (*wt*)) and *katG* (S315T) (MIC (*katG* S315T)) strains of *Mtb*.

Compound	IC_{50} /μg mL^{-1}	MIC (*wt*)/μg mL^{-1}	MIC (*katG* S315T) /μg mL^{-1}
INH	> 27.4	0.04	6.0
INH-C_{10}	> 7.3	0.11	2.0
N33	> 7.1	0.3	6.0
N34	> 63.5	0.3	6.0
N33red	> 57.1	0.6	> 37
N34red	15.5	0.3	> 41

Table S2 – HSA binding constants of the compounds studied in this work retrieved from the variation in the steady-state fluorescence intensity (K_b), from the variation in the fluorescence intensity which is not accounted for by the decrease in fluorescence lifetime (K'_b) and from the variation in $\bar{\tau}$, the amplitude-weighted mean fluorescence lifetime (K''_b). The binding constants of the compounds for the warfarin binding site in the presence of equimolar proportions of HSA and the competitor warfarin (K_{bc}) are also shown.

Compound	10^{-3} K_b / M^{-1}	10^{-3} K'_b / M^{-1}	10^{-3} K''_b / M^{-1}	10^{-3} K_{bc} / M^{-1}
INH	0.37 ± 0.01	0.37 ± 0.01	-	0.22 ± 0.01
INH-C_{10}	21.8 ± 4.48	18.6 ± 5.58	8.33 ± 0.21	2.43 ± 0.86
N33	6.04 ± 1.75	5.00 ± 2.50	12.5 ± 9.4	18.5 ± 1.3
N34	7.94 ± 2.93	7.69 ± 3.68	1.92 ± 0.07	7.8 ± 0.3
N33red	1.77 ± 0.65	1.77 ± 0.65	-	1.34 ± 0.67
N34red	6.69 ± 1.28	6.25 ± 2.29	1.37 ± 0.11	2.99 ± 0.17
